# Air pollution associated with hospital visits for mental and behavioral disorders in Northeast China

**DOI:** 10.3389/fepid.2023.1090313

**Published:** 2023-03-30

**Authors:** Huo Liu, Hang Zhao, Jinling Huang, Miao He

**Affiliations:** ^1^ Liaoning Key Laboratory of Environmental Health Damage Research and Assessment, Department of Environmental Health, School of Public Health, China Medical University, Shenyang, China; ^2^ Department of Hospital Management Office, Shengjing Hospital of China Medical University, Shenyang, China; ^3^ Department of Cardiology, Shengjing Hospital of China Medical University, Shenyang, China

**Keywords:** air pollution, mental and behavioral disorders, hospital visits, short-term exposure, time series

## Abstract

**Background:**

Related studies have found that air pollution is an important factor affecting mental and behavioral disorders. Thus, we performed this time-series study to evaluate the relationship between short-term exposure to ambient air pollutants and visits to hospital by patients with mental and behavioral disorders in northeastern China.

**Methods:**

We used quasi-Poisson regression models and generalized additive models to probe the links between air pollution and mental and behavioral disorders. The possible influences were also explored stratified by season, age and gender.

**Results:**

We found that sulfur dioxide (SO_2_) had a cumulative effect on mental and behavioral disorders at lag04–lag07 and had the greatest effect at lag07 [Relative risk (RR) = 1.068, 95%CI = 1.021–1.117]. Particulate matter of size 2.5 μm (PM_2.5_) and SO_2_ had a cumulative effect on depression and both had the largest effect at lag07 (RR = 1.021, 95%CI = 1.002–1.041; RR = 1.103, 95%CI = 1.032–1.178); SO_2_ also had a cumulative effect on anxiety disorders, with the largest effect at lag06 (RR = 1.058, 95%CI = 1.009–1.110). In the stratified analysis, people are more susceptible in the cold season compared to the warm season and females and the 18–60-year age group are more sensitive to air pollutants. It is suggested to strengthen management and preventive measures to decrease air pollution exposure.

**Conclusion:**

This study found an association between increased concentrations of air pollutants and increased outpatient visits for mental and behavioral disorders. We recommend that preventive and protective measures should be strengthened in an effort to reduce exposure to air pollution in order to maintain physical and mental health.

## Introduction

Mental and behavioral disorders such as anxiety disorder, depression and schizophrenia are a group of disorders in which the patient's brain functions are disturbed and cognitive and behavioral functions are impaired under the influence of various factors (e.g., biological, social and environmental) (1). This is not limited to a small group of susceptible individuals but is a major public health problem with significant consequences for society. According to the latest Global Burden of Disease estimates, in the 10–24-year age group depression and anxiety disorders accounted for the fourth and sixth highest rates of all disability-adjusted life years (2). Also, in the first national survey of mental disorders in China we found that anxiety disorders were the most prevalent lifelong disorder, with a prevalence of 7.6% for lifetime (3).

There are many risk factors for mental and behavioral disorders. Besides the known influence of genetic and biological factors (4, 5), socioeconomic and lifestyle factors on mental disorders (6), recent studies have shown that physical environmental factors (7, 8), especially air pollution, have become a factor that cannot be ignored when studying the causes of mental disorders (9). A Chinese multi-city study showed that short-term increases in fine particulate matter of size 2.5 μm (PM_2.5_), inhalable particulate matter (PM_10_), sulfur dioxide (SO_2_), nitrogen dioxide (NO_2_) and ozone (O_3_) were associated with exacerbations of mental and behavioral disorders; NO_2_ in particular is a more severe health risk than the other pollutants (10). A study from Germany reported a relative risk of 1.010 (95%CI = 1.005–1.014) for depression and of 1.007 (95%CI = 1.000–1.014) for anxiety disorder when the maximum 8 h average ozone concentration exceeded 120 μg/m^3^ for more than 10 consecutive days (11).

According to the recently published Lancet Burden of Disease, ambient particulate matter pollution is by far one of the greatest global environmental risks. China is the leading developing country in the world, and its accelerated economic development and industrialization have led to a growing air pollution concern. Despite a wide range of government policies and regulations in place to limit air pollutant releases, China is still facing a serious air pollution problem (12). Meanwhile many studies have focused on cardiovascular and respiratory disorders, with only a minority examined the connection between air pollution and mental and behavioral disorders. Moreover, to our knowledge, there is very limited research on the relations between exposure to ambient air pollutants and mental and behavioral disorders in northeastern China. Therefore, we collected outpatient cases of mental and behavioral disorder admission visits in Shenyang from January 1, 2017 to December 31, 2019, with the aim of analyzing the acute and cumulative lagged effects of air pollution on mental and behavioral disorder visits after controlling the long-term trends, week effects and the effects of meteorological factors. Furthermore, we also examined the correlation between the air pollutants and disease visits at different season, age and gender.

## Methods

**Figure 1:**
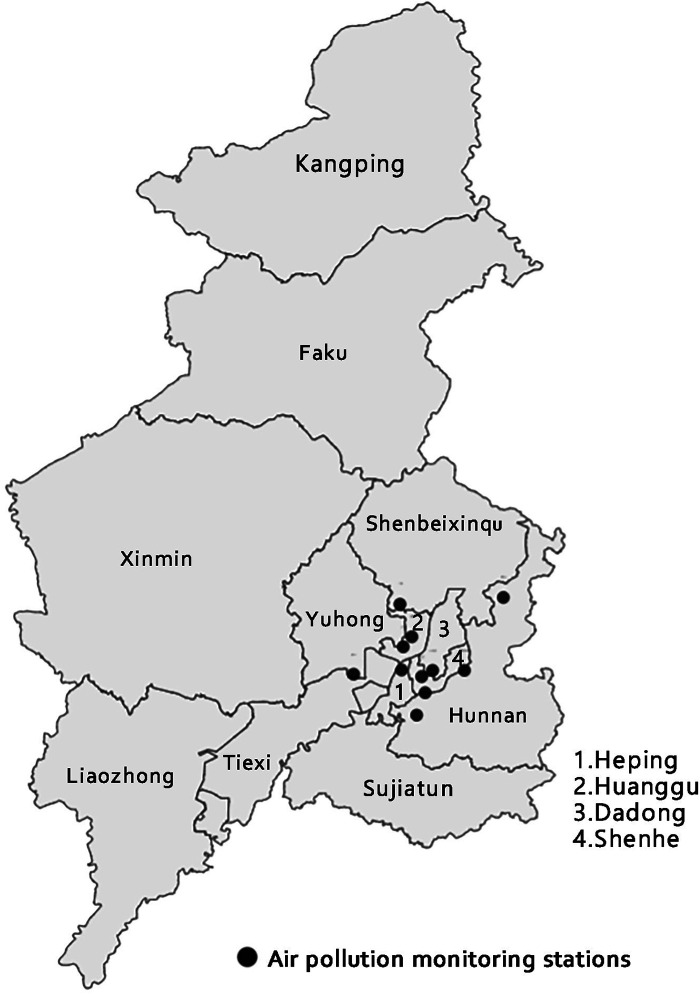
Distribution of air quality monitoring stations in Shenyang.

Shenyang is the largest city in Northeast China, with a total area of 12,860 km^2^. We collected 36,104 visits to a tertiary care hospital in Shenyang for mental and behavioral disorders from January 1, 2017 to December 31, 2019. The distribution map is shown in Figure 1. The case information collected mainly including the date of admission, age, gender, number of patients, preliminary diagnosis and the ICD-10 (International Classification of Diseases, 10th Revision) discharge diagnosis code. Mental and behavioral disorders were defined according to the ICD-10 with codes F01–F99. We considered two further categories of mental and behavioral disorders: depression (F32–F33) and anxiety disorder (F40–F41).

Air pollutant data (including levels of PM_2.5_, PM_10_, SO_2_, NO_2_, carbon monoxide (CO) and O_3_) in the study period come from 11 meteorological monitoring stations of the Liaoning Department of Environmental Protection, which have uniform construction standards and can better represent the air pollution concentration in Shenyang. We collected daily 24 h average PM_2.5_, PM_10_, SO_2_, NO_2_ and CO concentrations and 8 h sliding average O_3_ concentrations for each monitoring station from January 1, 2017 to December 31, 2019. After that, the daily average concentrations of PM_2.5_, PM_10_, SO_2_, NO_2_ and CO and the 8 h sliding average concentrations of O_3_ in Shenyang were derived from the measured data of 11 monitoring stations during the study period. Meteorological data, including daily average temperature and relative humidity, were acquired from the Liaoning Provincial Meteorological Bureau.

Summarizing the features of the study population, describing the air pollution and meteorological data, and stratifying them by cold and warm seasons. Due to the fact that the concentration distribution of pollutants does not conform to a normal distribution, Spearman's correlation was used to calculate the correlation between each pollutant and the meteorological factors in this study. We found mental and behavioral disorders admission follow an over-dispersed Poisson distribution, quasi-Poisson regression model was used in a generalized additive model to explore the association between air pollution and hospital visits for mental and behavioral disorders (13). To controlling potential confounding factors, we added few covariates to the primary model, based on former research: (1) the degree of freedom (df) of time was set to 6 to control long-term and temporal trends based on Akaike Information Code (14); (2) the df for temperature and humidity are set to 4 and 3, respectively, to eliminate the potential influence of meteorological factors; and (3) DOW and Holiday are the dummy viable of day of week and public holiday. In brief, the specific analytical model for this study can be expressed as follows:

$$\begin{array}{rl} \log[E(Y_{t})]=\; & \alpha +\beta x_{t}+\mathrm{ns}\;(\mathrm{Time},\;\mathrm{df}=6)+\mathrm{ns}\;(\mathrm{Temperature},\;\\ & \mathrm{df}=4)+\mathrm{ns}\;(\mathrm{Humidity},\;\mathrm{df}=3)+\text{Factor }(\mathrm{DO}W_{t})\\ & \;+Factor\;(Holiday) \end{array}$$

where *E*(*Y_t_*) represents the expected of mental and behavioral disorders on day *t*; *α* is the intercept distance; *β* shows the coefficient; *X_t_* indicates the daily average concentration of air pollutants; ns() represents the natural cubic spline function; df denotes the degrees of freedom; Time indicates the long-term trend and seasonality; Temperature is the daily average temperature (°C); Humidity is the daily average relative humidity (%); and DOW is a dummy variable used to control for the “day of the week effect”. In the model, a smoothing spline function is used to plot the exposure– response curves for each pollutant and disease.

Due to the lagged impact of air pollution on human health, this research analyzed the single-day lagged effect of air pollutants over 0–7 days (lag0–lag7) and the cumulative lagged effect over 0–7 days (lag01–lag07; the average of the present day and previous 1–7 days), fitting a separate model with the same parameter settings as the main model for each lag day.

In addition, stratified analyses were performed to explore the underlying effects on season (cold season: November to April; warm season: May to October), age group (<18, 18–60 and >60 years) and gender (female and male). Differences between stratified effect estimates were compared using *z*-tests (15). Finally, to assess the robustness of the model, we conducted a series of sensitivity analyses, which mainly covered two aspects: to control for the stability of the time trend, we vary the df of the time-smoothing function to 6–10 per year; and we established a two-pollutant model to test the validity of the effects estimates after controlling for co-contaminants.

Results are expressed by using the RR (and 95%CI) of mental and behavioral disorders per 10 µg/m^3^ air pollutant concentration increment. All analysis results were calculated using *R* statistical software (Version 1.4.1106). All the statistical tests were two-sided and differences were statistically significant at *P* < 0.05.

## Result

Between January 1, 2017 and December 31, 2019 a total of 36,104 patients with mental and behavioral disorders were admitted to the hospital (10,115 for depression and 15,991 for anxiety disorders) (Table 1). The average age of the study participants was 34.94 years. Among them, 13,720 (38%) were male and 22,384 (62%) were female. The number of visits for mental and behavioral disorders is a little higher during the warm season compared to the cool season (18,620 vs. 17,484).

**Table 1:** Characteristics of daily hospital admissions for mental and behavioral disorders in Shenyang from 2017 to 2019.

Variable	*n* (%)
Total number of admissions	36,104 (100%)
Mental and behavioral disorders	
Anxiety	15,991 (44.3%)
Depression	10,115 (28.0%)
Others	9,998 (27.7%)
Age, mean (SD)	34.94 (18.23)
<18	6,831 (18.9%)
18–60	25,072 (69.4%)
>60	4,201 (11.6%)
Gender	
Female	22,384 (62%)
Male	13,720 (38%)
Season	
Cold season	17,484 (48.4%)
Warm season	18,620 (51.6%)

Note: SD, standard deviation.

Descriptive statistics of air pollutants and meteorological variables are given in Table 2. Daily average concentrations (Standard deviation, SD) of air pollutants in Shenyang during the study period were 43.98 (32.04) μg/m^3^ for PM_2.5_, 79.1 (45.12) μg/m^3^ for PM_10_, 26.28 (20.9) μg/m^3^ for SO_2_, 36.9 (14.92) μg/m^3^ for NO_2_, 950 (430) μg/m^3^ for CO and 59.92 (35.25) μg/m^3^ for O_3_, separately. The daily mean temperature was 9.36°C and the mean relative humidity was 58.39%. The results of air pollutants stratified by season are shown in Table 3, which shows that the concentrations of all pollutants except ozone are higher in the cold season than in the warm season. Table 4 shows the correlations for each air pollutant and meteorological factors. It can be seen that PM_2.5_, PM_10_, SO_2_, NO_2_ and CO are highly correlated with each other (0.60 < *r* < 0.89) and yet all are negatively correlated with temperature (−0.57 < *r* < −0.18). In contrast, O_3_ was negatively correlated with PM_2.5_, PM_10_, SO_2_, NO_2_ and CO (−0.50 < *r* < −0.08) but positively correlated with temperature (*r* = 0.69).

**Table 2:** Descriptive statistics of statistics of daily environment data in Shenyang from 2017 to 2019.

Variable	mean	SD	Minimum	25th	50th	75th	Maximum
Air pollutants							
PM_2.5_ (μg/m^3^)	43.98	32.04	5	22	35	55	224
PM_10_ (μg/m^3^)	79.1	45.12	14	48	68	98	450
SO_2_ (μg/m^3^)	26.28	20.9	4	13	21	31	160
NO_2_ (μg/m^3^)	36.9	14.92	9	26	34	46	109
CO (μg/m^3^)	950	430	280	640	850	1160	2690
O_3_ (μg/m^3^)	59.92	35.25	9	33	54	80	218
Weather factors							
Temperature (°C)	9.36	13.17	−22.8	−2.6	11.3	21.3	32
Humidity (%)	58.39	17.23	16	45	59	71.8	96.8

Note: PM_2.5_ fine particles matter, PM_10_ inhalable particles matter, SO_2_ sulfur dioxide, NO_2_ nitrogen dioxide, CO, carbon monoxide, O_3_ ozone.

**Table 3:** Descriptive statistics of statistics of daily environment data in Shenyang from 2017 to 2019 after seasonal stratification.

Variable	mean	SD	Minimum	25th	50th	75th	Maximum
Cold Season							
PM_2.5_ (μg/m^3^)	56.93	36.54	10	31	47	72	224
PM_10_ (μg/m^3^)	95.34	47.11	25	60	86	119	450
SO_2_ (μg/m^3^)	35.66	24.79	6	20	29	43	160
NO_2_ (μg/m^3^)	41.86	14.94	13	30	41	52	85
CO (μg/m^3^)	1,051.43	473.61	360	670	950	1330	2680
O_3_ (μg/m^3^)	45.97	26.92	9	24	39	61	158
Temperature (°C)	−1.40	9.00	−22.8	−8.2	−2.9	4.9	21.8
Humidity (%)	50.30	14.70	16	39	50.5	60	91
Warm Season							
PM_2.5_ (μg/m^3^)	30.44	18.58	5	19	26	37	122
PM_10_ (μg/m^3^)	62.11	35.80	14	39	53	75	348
SO_2_ (μg/m^3^)	16.45	7.89	4	10	15	21	60
NO_2_ (μg/m^3^)	31.69	13.01	9	23	29	38	109
CO (μg/m^3^)	848.21	340.24	280	620	790	1000	2690
O_3_ (μg/m^3^)	74.52	37.00	15	44	71	95	218
Temperature (°C)	19.86	6.40	1	16.15	21.10	24.50	32.40
Humidity (%)	66.28	15.83	22.3	57	69	77.9	96.8

**Table 4:** Spearman correlation coefficients of air pollutants and meteorological factors.

	PM_2.5_	PM_10_	SO_2_	NO_2_	CO	O_3_	Temperature	Humidity
PM_2.5_	1.00							
PM_10_	0.89*	1.00						
SO_2_	0.73*	0.64*	1.00					
NO_2_	0.70*	0.60*	0.71*	1.00				
CO	0.79*	0.66*	0.71*	0.72*	1.00			
O_3_	−0.16*	−0.08*	−0.37*	−0.50*	−0.21*	1.00		
Temperature	−0.38*	−0.34*	−0.57*	−0.44*	−0.18*	0.69*	1.00	
Humidity	−0.09*	−0.28*	−0.18*	0.01	0.21*	−0.04	0.40*	1.00

Note: *P < 0.05.

**Figure 2:**
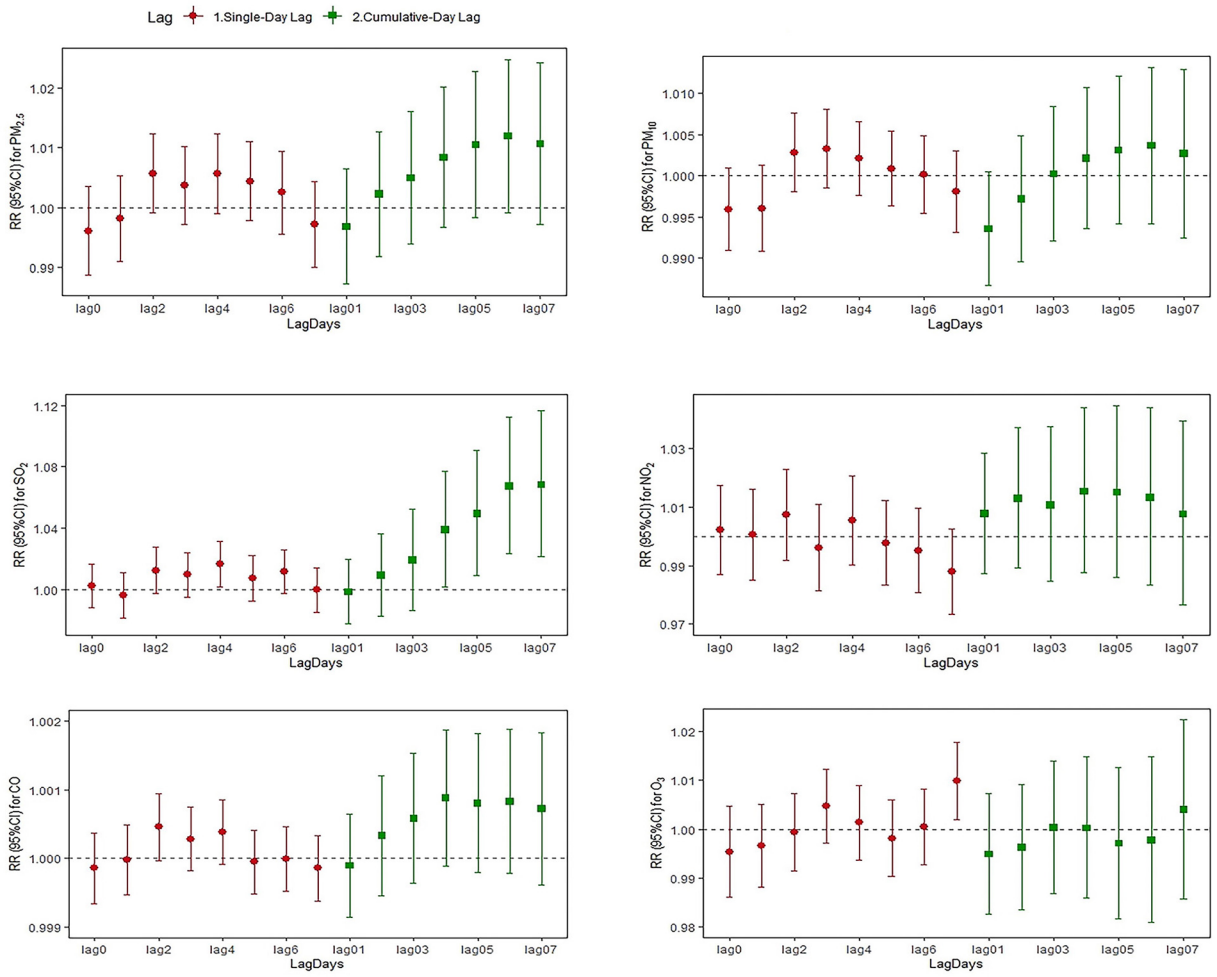
Relative risk and 95% CI of hospital admissions for mental and behavioral disorders at different lag days for every 10 µg/m^3^ increase in pollutant concentration.

**Figure 3:**
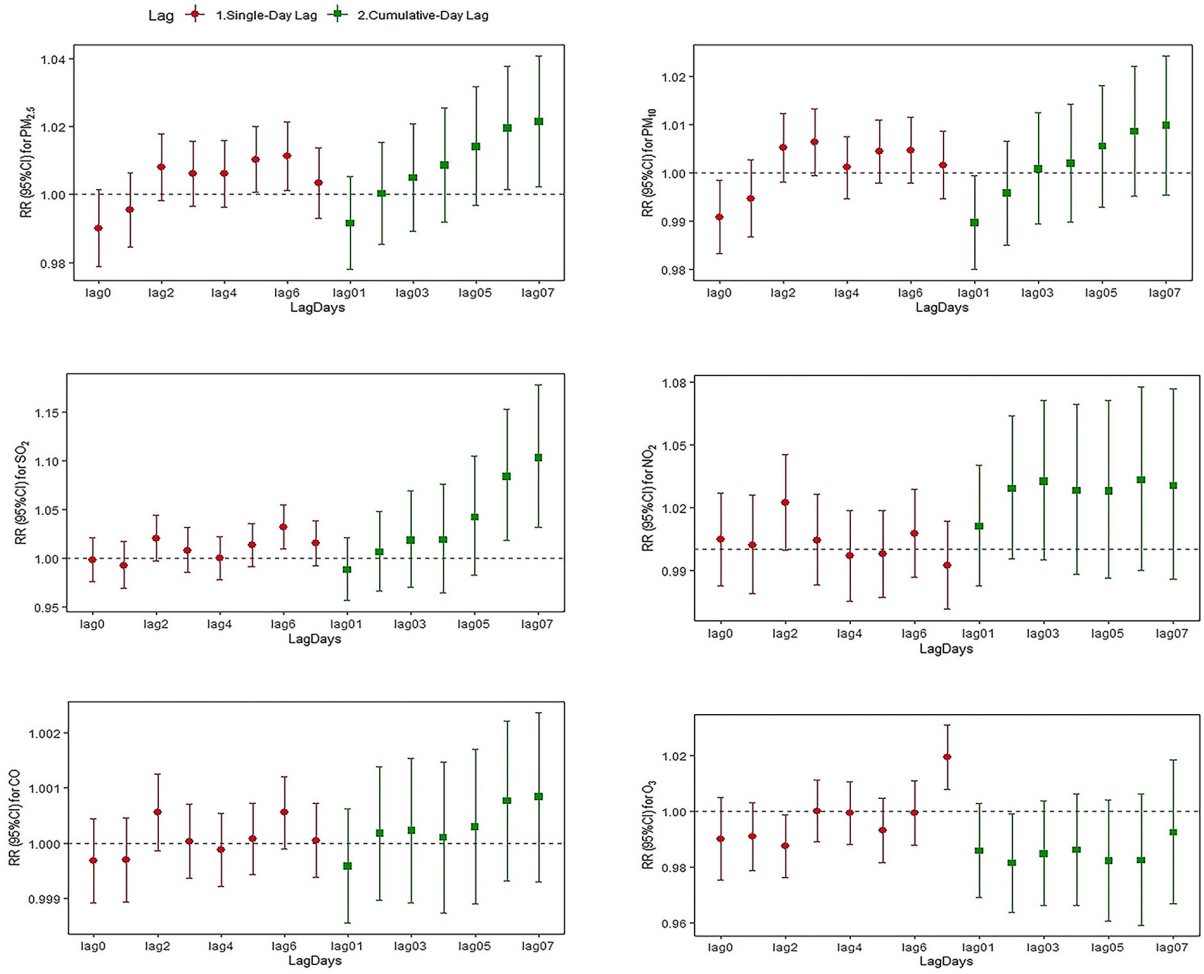
Relative risk and 95% CI of hospital admissions for depression at different lag days for every 10 µg/m^3^ increase in pollutant concentration.

**Figure 4:**
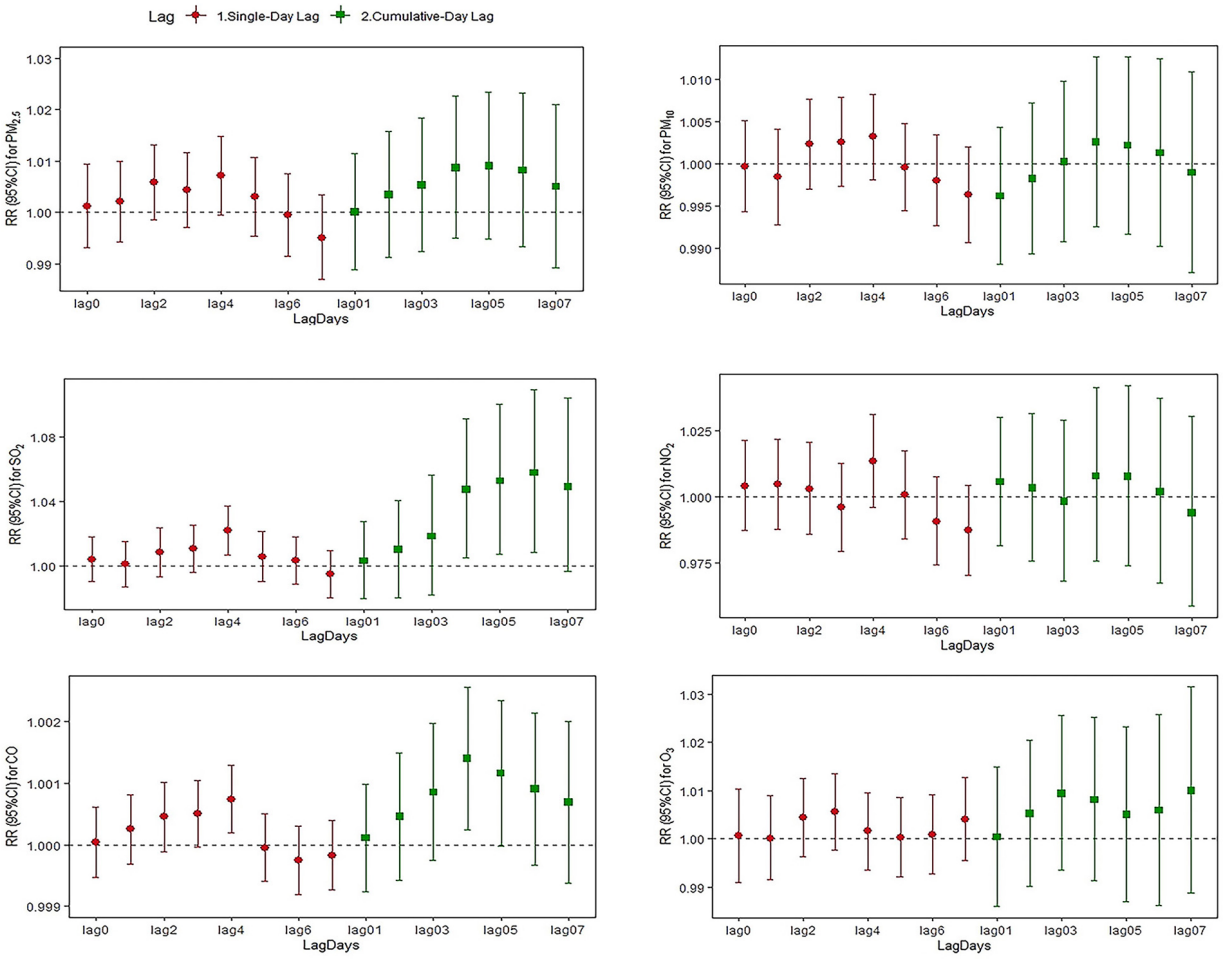
Relative risk and 95% CI of hospital admissions for anxiety at different lag days for every 10 µg/m^3^ increase in pollutant concentration.

Figures 2–4 and Supplementary Tables S1–S3 summarize the effect of mental and behavioral disorders, depression and anxiety disorders, respectively, on outpatient admissions in relation to air pollutants in a single-pollutant model. After adjusting for the long-term trend, meteorological elements and weekend effects, the effects of SO_2_ on mental and behavioral disorders at lag4 (RR = 1.016, 95%CI = 1.002–1.031) and O_3_ at lag7 (RR = 1.010, 95%CI = 1.002–1.018) were statistically significant for single-day effects. Among the cumulative effects, the effect of SO_2_ at lag04–lag07 for four continuous days was statistically significant, and the effect is strongest at lag07 (RR = 1.068, 95%CI = 1.021–1.117).In the single-day effect of air pollutants on depression, PM_2.5_ was statistically significant at lag5–lag6 (RR = 1.010, 95%CI = 1.001–1.020; RR = 1.011, 95%CI = 1.001–1.021) and O_3_ was statistically significant at lag 7 (RR = 1.019, 95%CI = 1.008–1.031). In the cumulative effect, both PM_2.5_ and SO_2_ were statistically significant at lag06–lag07 (for PM_2.5_: RR = 1.019, 95%CI = 1.001–1.038; RR = 1.021, 95%CI = 1.002–1.041; for SO_2_: RR = 1.083, 95%CI = 1.018–1.152; RR = 1.103, 95%CI = 1.032–1.178). For anxiety disorders, both SO_2_ and CO were statistically significant at lag4 (for SO_2_: RR = 1.022, 95%CI = 1.007–1.037; for CO: RR = 1.001, 95%CI = 1.000–1.001). Meanwhile, SO_2_ was statistically significant at lag04–lag06 (RR = 1.047, 95%CI = 1.005–1.091; RR = 1.053, 95%CI = 1.008–1.100; RR = 1.058, 95%CI = 1.009–1.110).

Stratified analysis by season, age and gender groups is shown in Supplementary Figures S1–S9. After seasonal stratification (Supplementary Figures S1–S3 and Tables S4–S6), SO_2_, NO_2_ and CO had cumulative effects on mental and behavioral disorders during the cold season. All five pollutants except O_3_ had a significant effect on depression. Specifically, PM_2.5_ had a single-day lagged effect on depression on all consecutive five days during lag2–lag6, and the effect was highest at lag5 (RR = 1.020, 95%CI = 1.009–1.032). Meanwhile, PM_2.5_, SO_2_, NO_2_ and CO also had an effect on anxiety during the cold season. In particular, SO_2_ had a cumulative effect on anxiety disorders during lag04–lag07 (RR = 1.060, 95%CI = 1.007–1.015; RR = 1.068, 95%CI = 1.010–1.130; RR = 1.078, 95%CI = 1.012–1.147; RR = 1.071, 95%CI = 1.000–1.147) for four consecutive days.

In the age-specific analysis (Supplementary Figures S4–S6 and Tables S7–S9), PM_10_ in lag2 (RR = 1.008, 95%CI = 1.001–1.015) had an impact on mental and behavioral disorders among people younger than 18 years. In the population aged 18–60 years, SO_2_ was significant at lag06–lag07 (RR = 1.049, 95%CI = 1.008–1.092; RR = 1.048, 95%CI = 1.004–1.094). PM_2.5_, PM_10_, SO_2_ and O_3_ had a significant effect on depression in patients aged 18–60 years, both PM_2.5_ and PM_10_ had an effect in lag07 (RR = 1.040, 95%CI = 1.001–1.082; RR = 1.031, 95%CI = 1.002–1.061) for people younger than 18 years. Also, SO_2_, CO and O_3_ had effects on patients with anxiety disorders in the 18–60-year age group. It is noteworthy that SO_2_ had an effect on all people older than 60 years.

In the gender analysis (Supplementary Figures S7–S9 and Tables S10–S12), we found a more pronounced effect of air pollutants on women, especially SO_2_ had an effect on all female patients. Furthermore, PM_2.5_ had an effect on female depressed patients on two consecutive days at lag5–lag6 (RR = 1.014, 95%CI = 1.002–1.025; RR = 1.012, 95%CI = 1.000–1.024). CO had an effect on female anxiety patients at lag4 (RR = 1.001, 95%CI = 1.000–1.001) and lag04 (RR = 1.002, 95%CI = 1.000–1.003). However, there was no significant difference between subgroups in the three-group stratified analysis.

The exposure response curves for air pollutants and mental and behavioral disorders can be seen in Supplementary Figures S10–S11. We found no significant threshold concentrations between air pollutants and mental and behavioral disorders. It can also be observed that the log relative risk of depression is almost linearly related to PM_2.5_, PM_10_ and SO_2_, with the risk increasing with increasing pollutant concentrations. The log relative risk of anxiety disorders increased with increasing PM_10_, SO_2_.

In the sensitivity analysis Supplementary Table S13 presents the results of air pollutants on mental and behavioral disorders at different degrees of freedom of the temporal smoothing function. For the temporal smoothing function, the estimated effect changed slightly at different degrees of freedom, suggesting that the findings on the connection between air pollutant exposure and outpatient visits for mental and behavioral disorders is robust in our study. In the two-pollutant model (Supplementary Table S13), the effects of PM_2.5_, SO_2_ and O_3_ on depression were stable after adjusting for other pollutants. Anxiety disorders effects of SO_2_ and CO also persist significant after adjusting for other pollutants.

## Discussion

To our knowledge, it is one of the few studies examining the relationship between air pollutants and mental and behavioral disorders in northeastern China. We used the outpatient data on mental and behavioral disorders in Shenyang during 2017–2019 and the conclusions of the time-series study showed that SO_2_ and O_3_ exposure were significantly related to increased risk of mental and behavioral disorders. Also, PM_2.5_, SO_2_ and O_3_ were significantly associated with depression and SO_2_ and CO were significantly associated with anxiety disorders. In subgroup analyses, the effects of the cold season were found to be greater than those of the warm season; furthermore, females and those aged 18–60 years were more vulnerable to pollutants. The possible mechanism is that air pollutants enter the central nervous system directly after inhalation through the mouth and nose, or enters the lungs and then through the body circulation to the brain parenchyma. Exposure to air pollutants leads to an increase in pro-inflammatory mediators and reactive oxygen species, with inflammation and oxidative stress in the brain possibly being a key link to air pollutants causing neurological disorders.

Notably, in our study we found that depression was always associated with SO_2_ and that anxiety was consistently associated with CO, relationships that persisted across the stratified analyses and two-pollutant models. Time series studies conducted in Xi'an and Ningbo, China, found the strongest correlation between SO_2_ with depression visits on the day of exposure (16, 17). A study including data on depression in 75 cities showed that the effects of air pollutants, excluding O_3_, were significant at several lags estimated over a 7-day period for depression. Meanwhile, the effects of NO_2_ were more robust in the subgroup, sensitivity and two-pollutant model analyses (18). However, a significant association between anxiety disorders with SO_2_ and NO_2_ was found in two other time series studies (19, 20). This variation may be attributed to differences in study populations, design protocols, local air pollutant levels, and meteorological factors. Therefore, it is necessary to further explore the link between air pollution and mental disorders.

Temperature is an important factor that affects the human mental condition (21). Shenyang is located in northeast China, with a large temperature difference between summer and winter, and the lowest temperature in winter can reach −22.8°C. Coal heating in winter can also lead to a large increase in pollutant concentrations. After a stratified analysis by season, we found that pollutants other than O_3_ tend to have a stronger effect on mental and behavioral disorders during the cold season compared to the warm season. In contrast, during the warm season O_3_ has a greater influence on people's depressed mood; this is concordant with the study of Zhang et al. (13, 22), which suggests that hypothermia has a more widespread and pervasive effect on this specific disease. However, Min et al. found that a high apparent environmental temperature is a risk contributor for mental disorders and that patient management services should be enhanced (23, 24). This variability may stem from different exposure patterns and the sensitivity of populations to the temperature in different regions.

In terms of age, we divided age into three stages and found that the association trend with mental and behavioral disorders was greater in the 18–60-year age group in our study compared to other age groups. This may be due to the fact that retirement, without the pressures of work, allows more time to pursue hobbies and leisure activities, thus alleviating mental health and other issues (25). A recent study also showed that people with poorer working conditions experienced substantial improvements in mental health problems after retirement, especially in the short term (26). With regard to gender, previous researches have revealed that the effect of air pollutants on mental and behavioral disorders tends to be more pronounced in females (16, 27), which is consistent with our findings. This may be due to the physiological differences between the sexes, including hormonal status, inflammatory response, differences in the size of their lungs and airway diameter, which affect particle deposition, patterns of breathing, airway resistance and exposure differences (28).

In the end there are some limitations of our study. Firstly, this study is an ecological study, which is the result of observations from a population, and the conclusions obtained cannot prove causality; we did not control for temperature lag, simply stratified the temperature. Secondly, due to the epidemic, the cut-off time of this study is early, and at the same time, the epidemic may have potential effects on the attendance behavior of the population and pollutant emissions that cannot be estimated, so the conclusions of this study are relevant for assessing the relationship between mental illness and air pollutants after the end of the epidemic are still relevant; finally, the composition of each air pollutant is complex, and we cannot determine which components of the air pollutant are the primary cause of the association.

## Conclusion

Our research shows that exposure to air pollutants on a short-term basis is related to an elevated risk of developing mental and behavioral disorders, and that this effect varies by season, age, and gender. The effect of air pollutants on disease also stayed robust after adjustment for other pollutants. Our findings provide support for exploring the connection between air pollution and mental disorders and for local policy makers to develop air control measures. Further research is required to confirm our discoveries and to investigate the potential mechanisms of this association.

## Data Availability

The original contributions presented in the study are included in the article/**Supplementary Material**, further inquiries can be directed to the corresponding author.
