# Supplementary material for: Air pollution associated with hospital visits for mental and behavioral disorders in Northeast China
Source: Front Epidemiol. 2023 Mar 30;3:1090313. doi: 10.3389/fepid.2023.1090313 (PMC10910900; doi:10.3389/fepid.2023.1090313)
Supplement: Supplementary file 1 [file Datasheet1.pdf]

Supplementary Material

Air pollution associated with hospital visits for mental and behavioral disorders in northeast China

Huo Liu, Hang Zhao, Jinling Huang, Miao He*
*Correspondence author

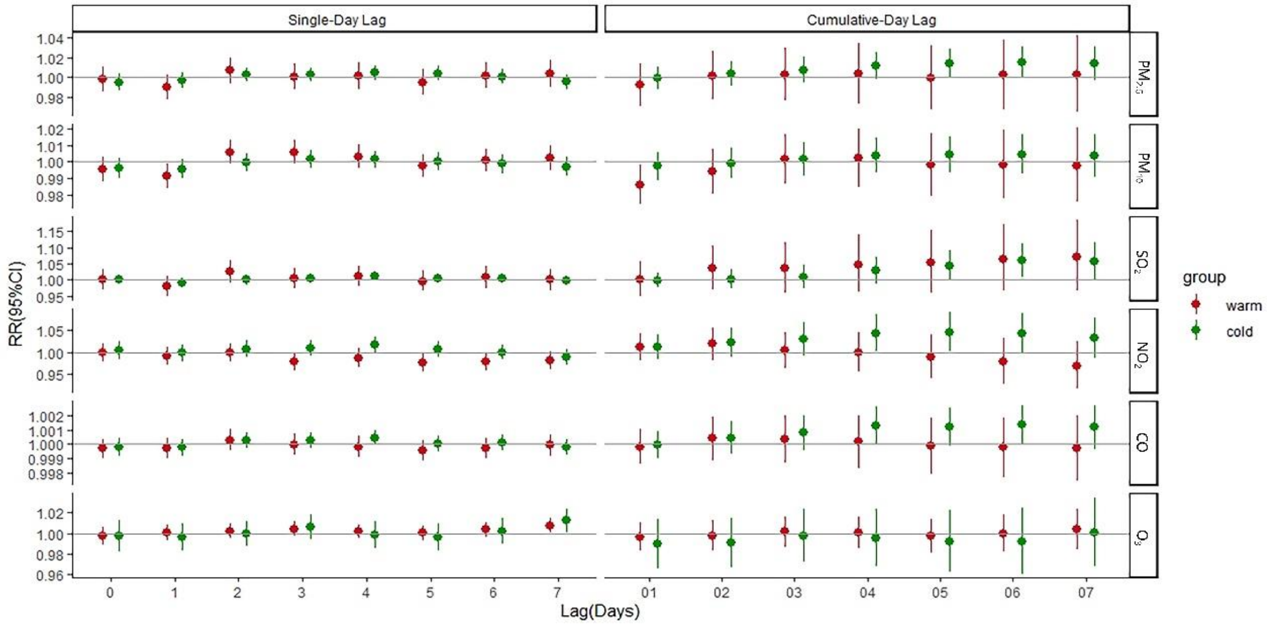

Figure S1. Relative risk and 95% CI of hospital admissions for mental and behavioral disorders atdifferent lag days for every 10 μg/m^3 increase in pollutants after seasonal stratification

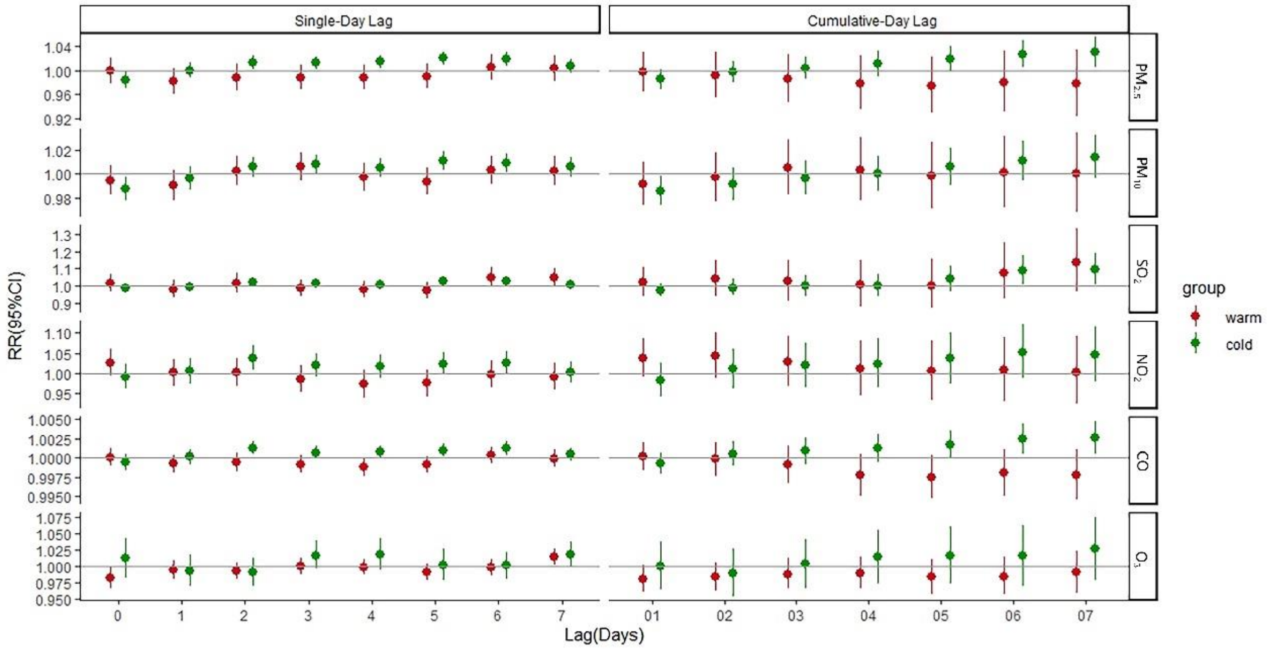

Figure S2. Relative risk and 95% CI of depression hospital admissions at different lag days for every 10 μg/m^3 increase in pollutants after seasonal stratification

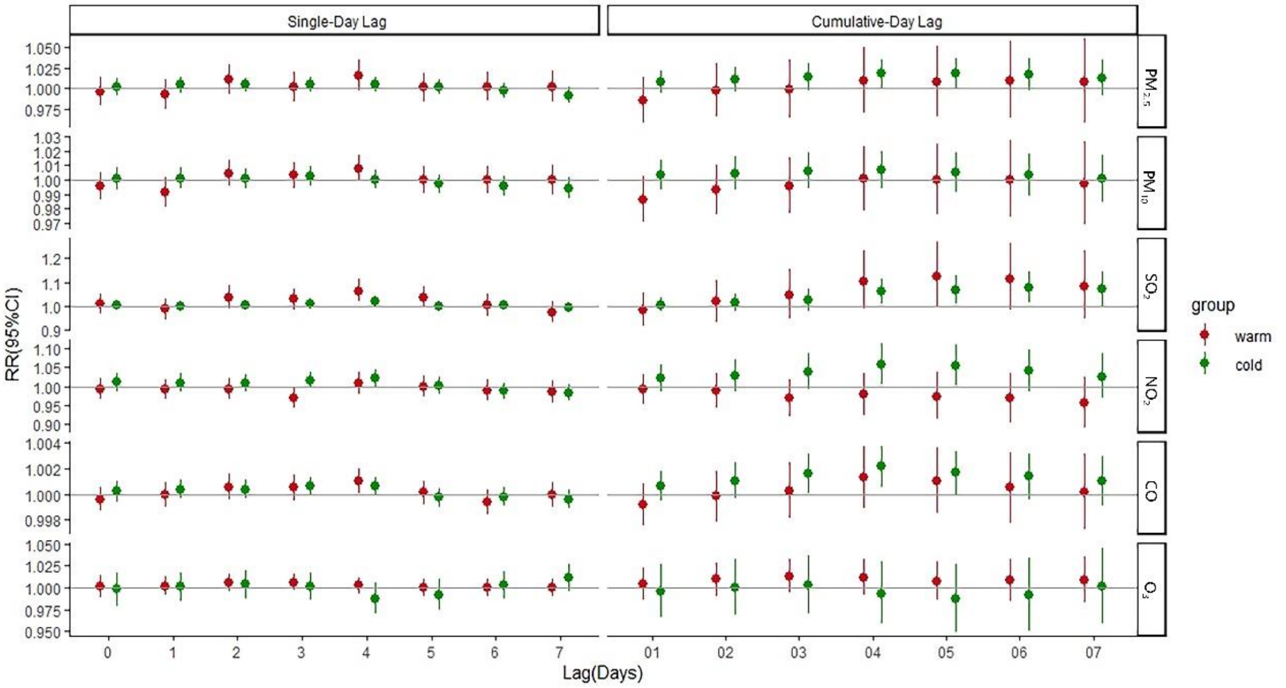

Figure S3. Relative risk and 95% CI of hospital admissions for anxiety disorders at different lag days for every 10 μg/m^3 increase in pollutants after seasonal stratification

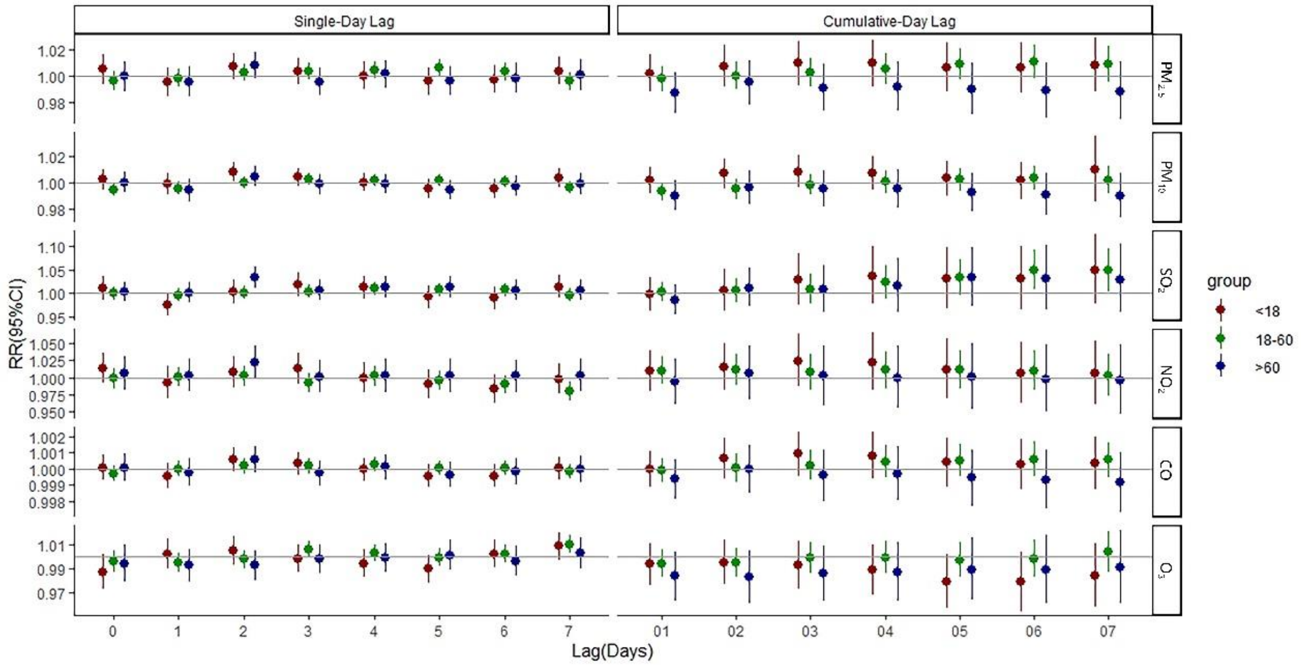

Figure S4. Relative risk and 95% CI of hospital admissions for mental and behavioral disorders at different lag days for every 10 μg/m^3 increase in pollutants after age stratification

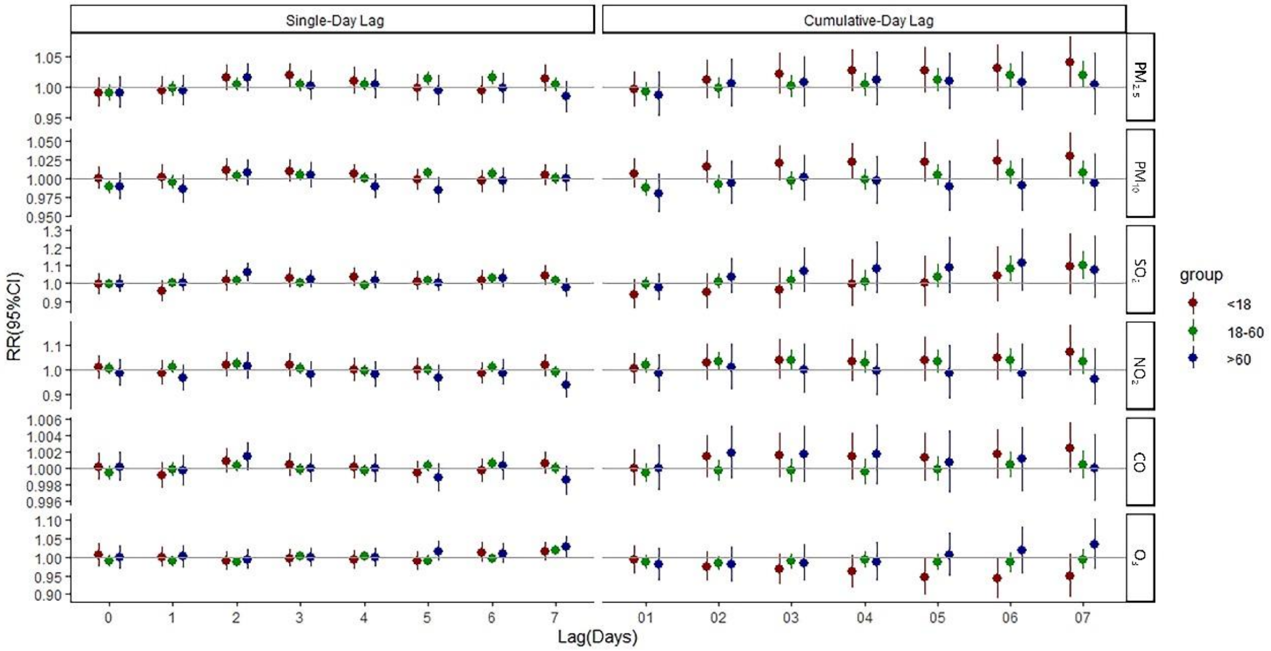

Figure S5. Relative risk and 95% CI of depression hospital admissions at different lag days for every 10 μg/m^3 increase in pollutants after age stratification

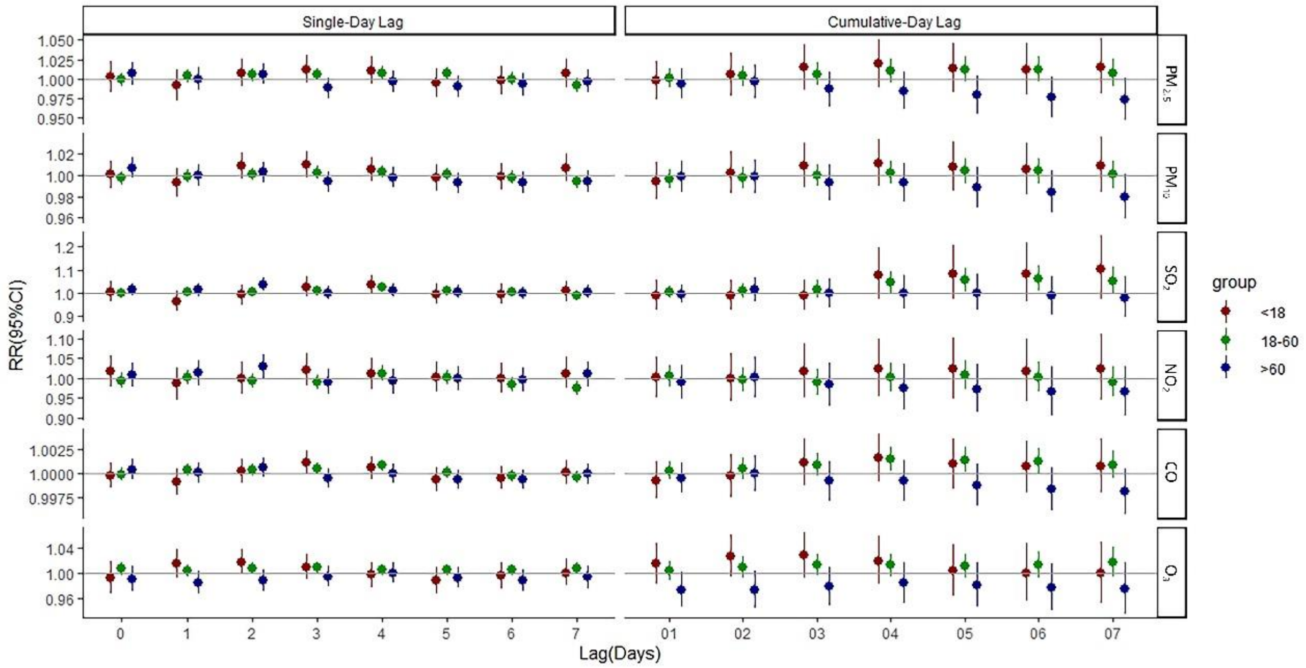

Figure S6. Relative risk and 95% CI of hospital admissions for anxiety disorders at different lag days for every 10 μg/m^3 increase in pollutants after age stratification

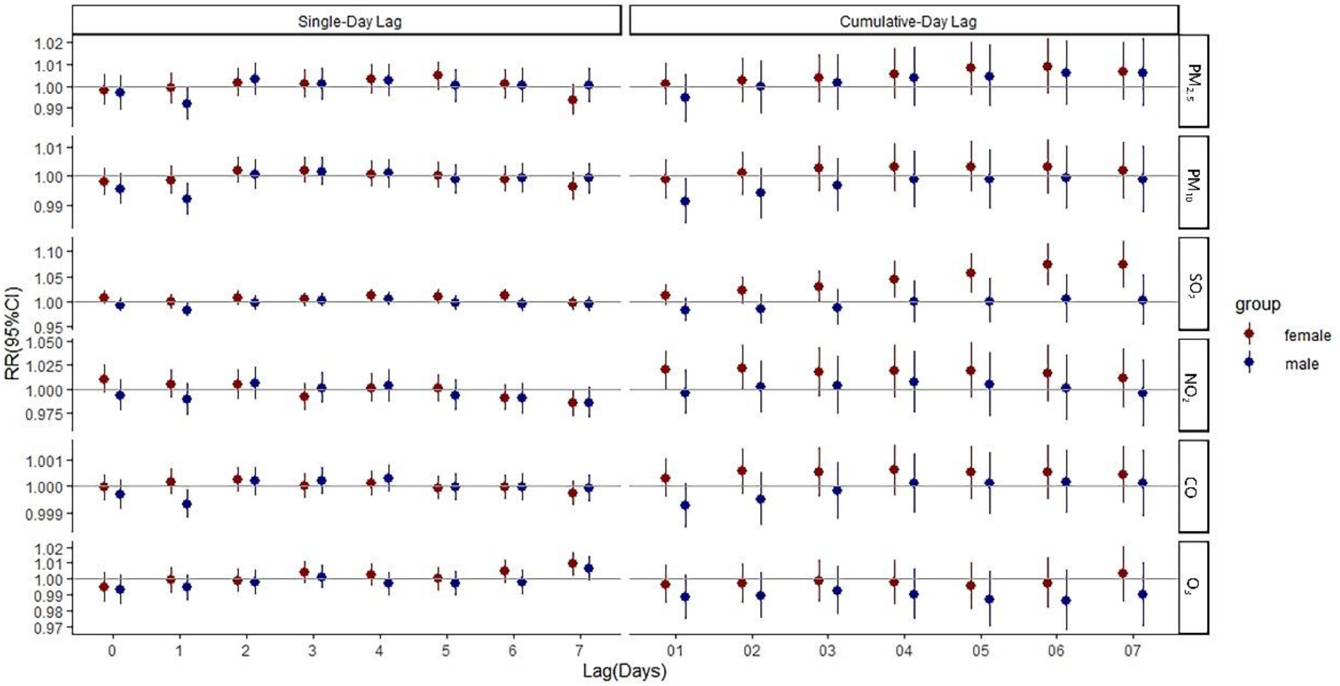

Figure S7. Relative risk and 95% CI of hospital admissions for mental and behavioral disorders at different lag days for every 10 μg/m^3 increase in pollutants after gender stratification

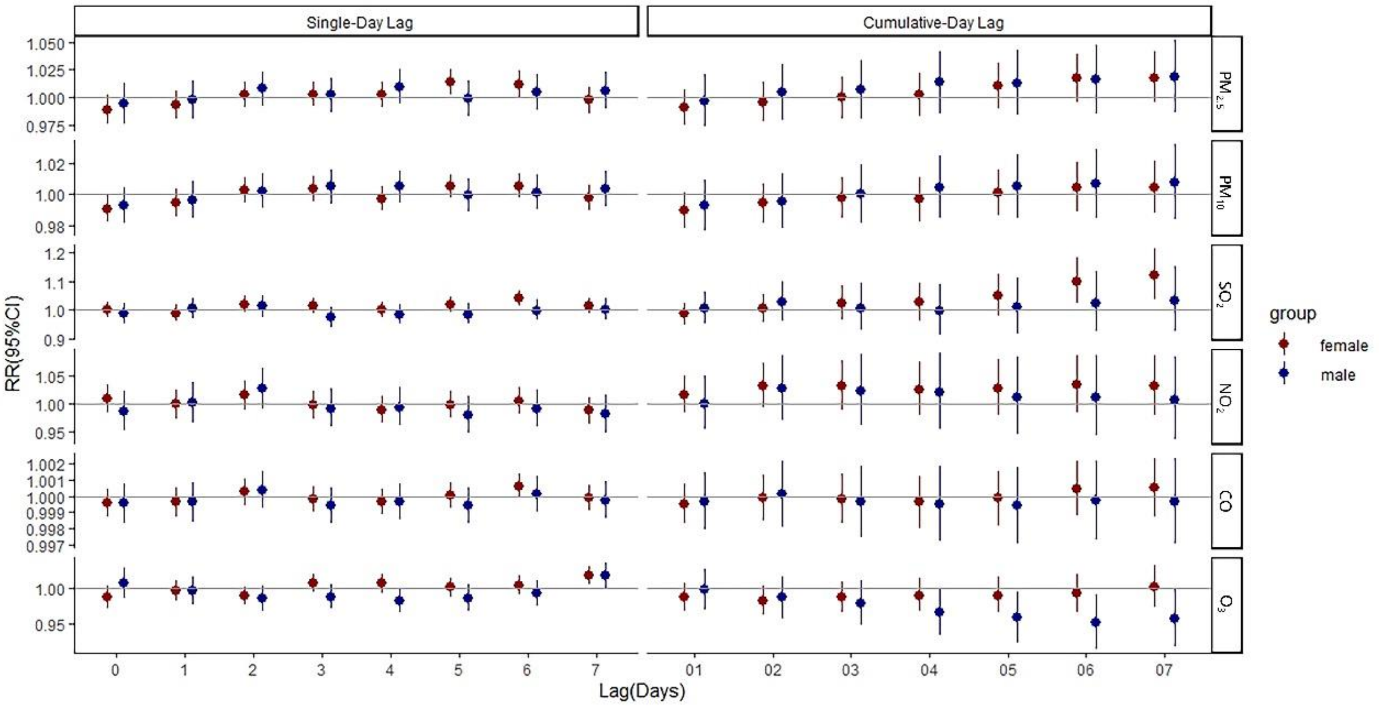

Figure S8. Relative risk and 95% CI of depression hospital admissions at different lag days for every 10 μg/m^3 increase in pollutants after gender stratification

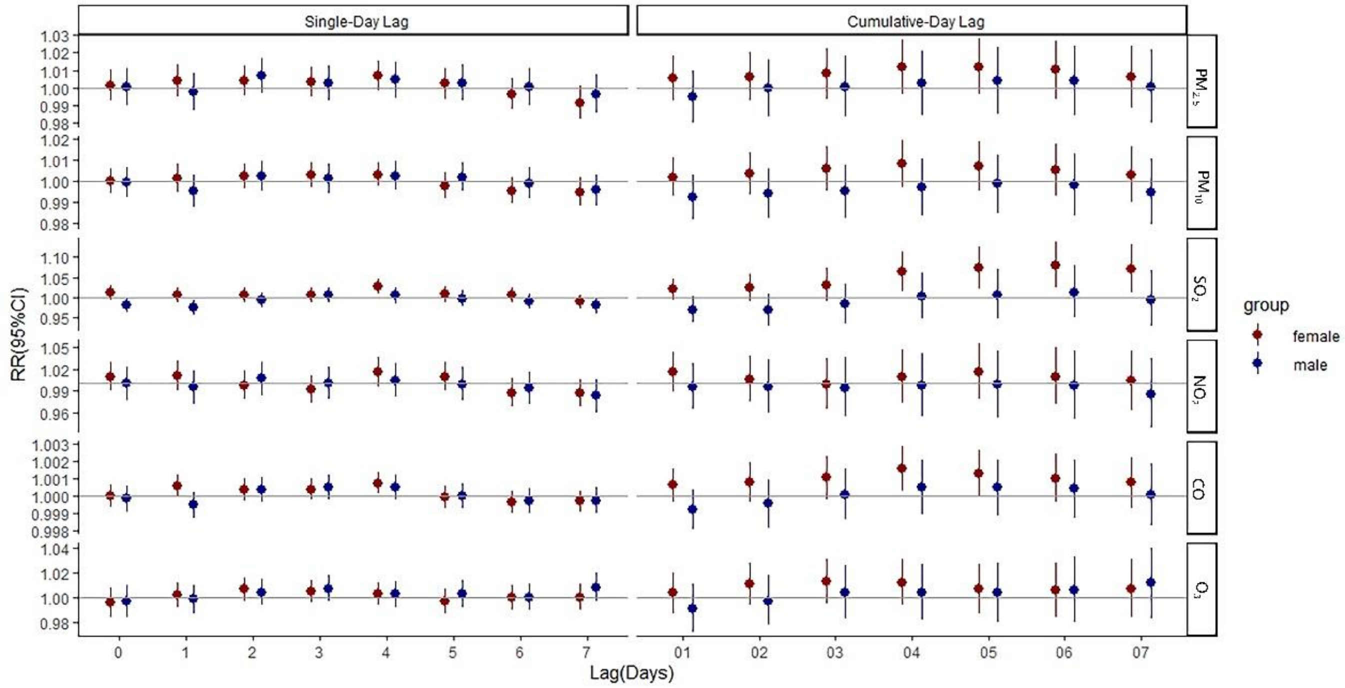

Figure S9. Relative risk and 95% CI of hospital admissions for anxiety disorders at different lag days for every 10 μg/m^3 increase in pollutants after gender stratification

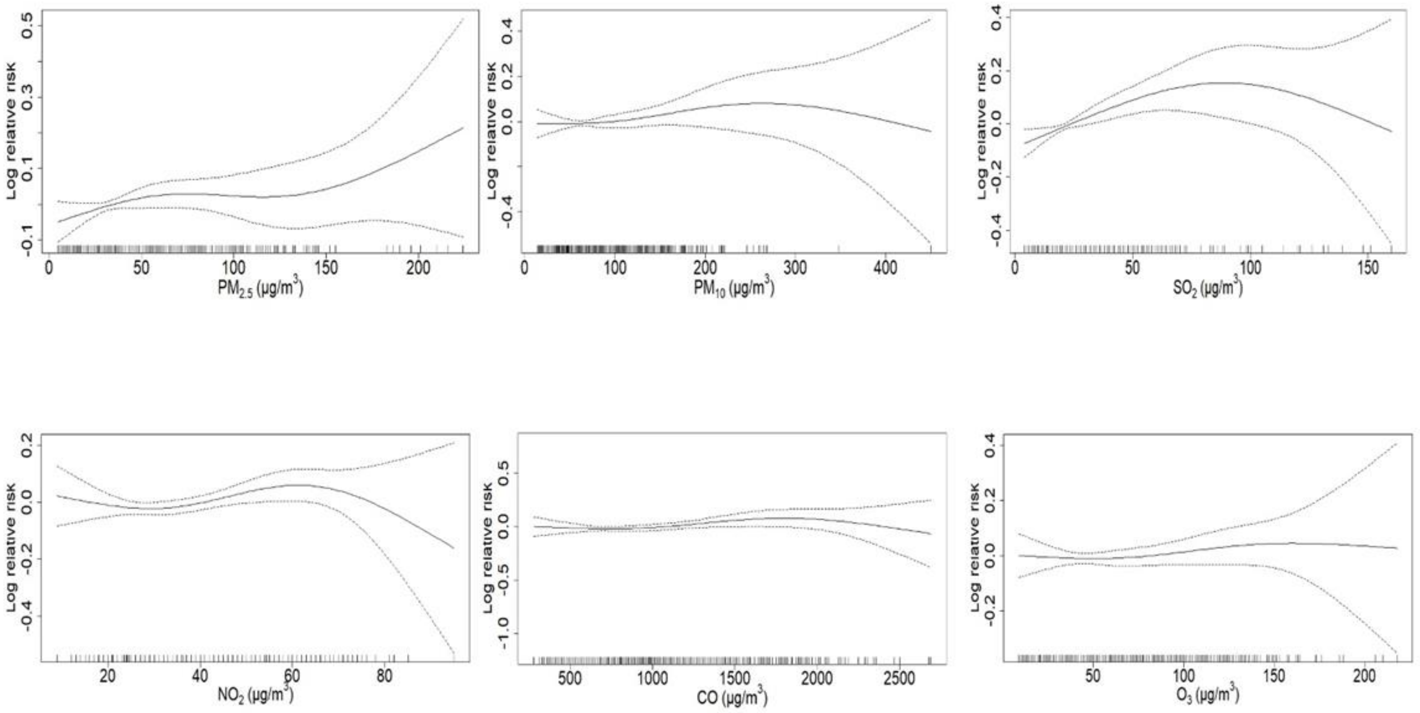

Figure S10. Exposure response curves for air pollutants and depression.

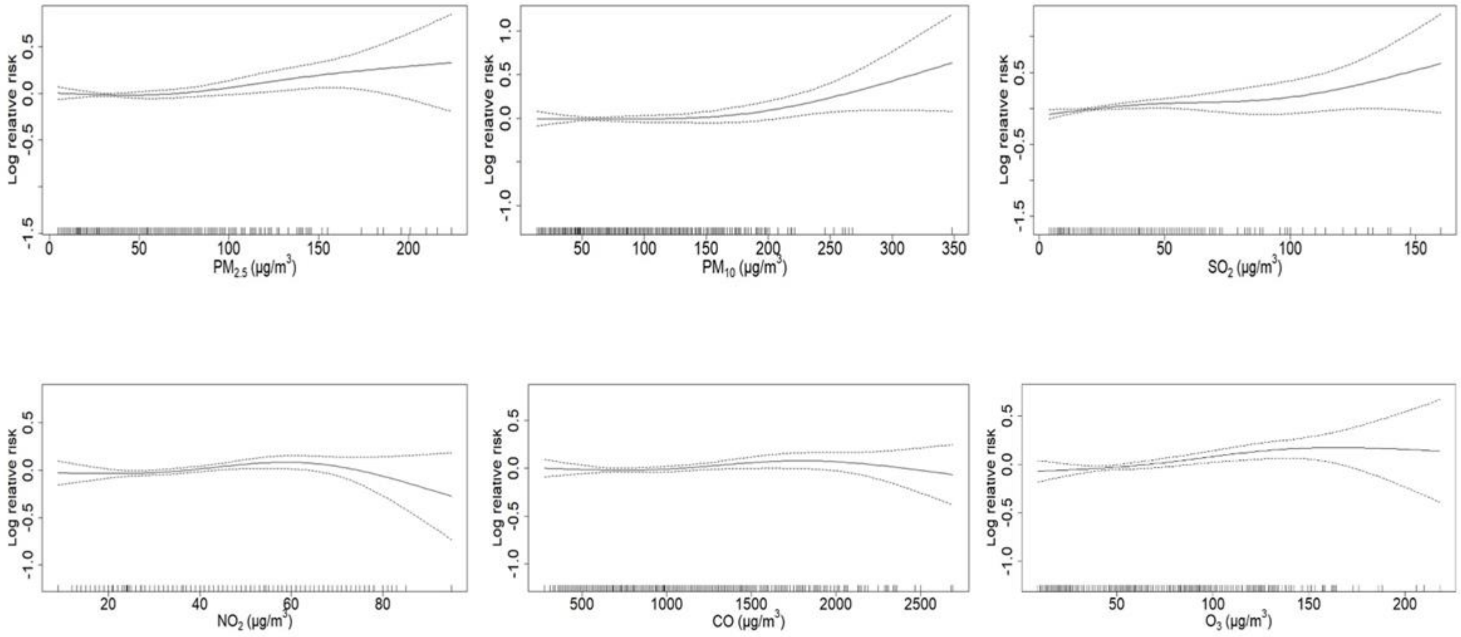

Figure S11. Exposure response curves for air pollutants and anxiety disorder.

Table S1. Relative risk and 95% CI of hospital admissions for mental and behavioral disorders at different lag days for every 10 μg/m^3 increase in pollutant concentration

	$PM_{2.5}$ RR(95%CI)	PM_{10} RR(95%CI)	SO_2 RR(95%CI)	NO_2 RR(95%CI)	CO RR(95%CI)	O_3 RR(95%CI)
lag0	0.996 (0.989,1.004)	0.996 (0.991,1.001)	1.002 (0.988,1.017)	1.002 (0.987,1.017)	1.000 (0.999,1.000)	0.995 (0.986,1.005)
lag1	0.998 (0,991,1.005)	0.996 (0.991,1.001)	0.996 (0.982,1.011)	1.000 (0.985,1.016)	1.000 (0.999,1.000)	0.997 (0.988, 1.005)
lag2	1.006 (0.999,1.012)	1.003 (0.998,1.008)	1.012 (0.997,1,027)	1.007 (0.992,1.023)	1.000 (1.000, 1.001)	0.999 (0.992, 1.007)
lag3	1.004 (0.997,1.010)	1.003 (0.999,1.008)	1.010 (0.995,1.024)	0.996 (0.982,1.011)	1.000 (1.000, 1.001)	1.005 (0.997, 1.012)
lag4	1.006 (0.999,1.012)	1.002 (0.998,1.008)	**1.016 (1.002,1.031)**	1.005 (0.990,1.021)	1.000 (1.000, 1.001)	1.001 (0.994, 1.009)
lag5	1.004 (0.998,1.011)	1.001 (0.996,1.005)	1.007 (0.993,1.022)	0.998 (0.983,1.012)	1.000 (0.999, 1.000)	0.998 (0.990, 1.006)
lag6	1.003 (0.996,1.009)	1.000 (0.995,1.005)	1.012 (0.997,1.026)	0.995 (0.981,1.009)	1.000 (0.999, 1.000)	1.000 (0.993, 1.008)
lag7	0.997 (0.990,1.004)	0.998 (0.993,1.003)	1.000 (0.985,1.014)	0.988 (0.973,1.002)	1.000 (0.999, 1.000)	**1.010 (1.002 , 1.018)**
lag01	0.997 (0.987,1.006)	0.994 (0.987,1.001)	0.988 (0.977 ,1.020)	1.008 (0.987,1.028)	1.000 (0.999, 1.001)	0.995 (0.983, 1.007)
lag02	1.002 (0.992,1.013)	0.997 (0.990,1.005)	1.009 (0.983,1.036)	1.013 (0.989,1.037)	1.000 (0.999, 1.001)	0.996 (0.984, 1.009)
lag03	1.005 (0.994,1.016)	1.000 (0.992,1.008)	1.019 (0.987,1.052)	1.011 (0.985,1.037)	1.001 (1.000, 1.002)	1.000 (0.987, 1.014)
lag04	1.008 (0.997,1.020)	1.002 (0.994,1.011)	**1.039 (1.002,1.077)**	1.015 (0.987,1.044)	1.001 (1.000, 1.002)	1.000 (0.986, 1.015)
lag05	1.010 (0.998,1.023)	1.003 (0.994,1.012)	**1.049 (1.009,1.091)**	1.015 (0.986,1.045)	1.001 (1.000, 1.002)	0.997 (0.982, 1.013)
lag06	1.012 (0.999,1.025)	1.004 (0.994,1.013)	**1.067 (1.024,1.113)**	1.013 (0.984,1.044)	1.001 (1.000, 1.002)	0.998 (0.981, 1.015)
lag07	1.011 (0.997,1.024)	1.003 (0.993,1.013)	**1.068 (1.021,1.117)**	1.007 (0.977,1.039)	1.001 (1.000, 1.002)	1.004 (0.986, 1.022)

Note: statistically significant ($P < 0.05$) are indicated in bold.

Table S2. Relative risk and 95% CI of depression hospital admissions at different lag days for every 10 μg/m³ increase in pollutant concentration

	$PM_{2.5}$ RR(95%CI)	PM_{10} RR(95%CI)	SO_2 RR(95%CI)	NO_2 RR(95%CI)	CO RR(95%CI)	O_3 RR(95%CI)
lag0	0.990 (0.979,1.002)	0.991 (0.983, 0.998)	0.999 (0.976, 1.022)	1.005 (0.983,1.027)	1.000 (0.999,1.000)	0.990 (0.975, 1.005)
lag1	0.995 (0.985,1.006)	0.995 (0.987, 1.003)	0.993 (0.969, 1.017)	1.002 (0.979 ,1.026)	1.000 (0.999,1.000)	0.991 (0.979, 1.003)
lag2	1.008 (0.998, 1.018)	1.005 (0.998, 1.012)	1.020 (0.997, 1.044)	1.022 (0.999, 1.046)	1.001 (1.000,1.001)	0.987 (0.976, 0.999)
lag3	1.006 (0.997, 1.016)	1.006 (0.999, 1.013)	1.008 (0.986, 1.031)	1.004 (0.983, 1.026)	1.000 (0.999, 1.001)	1.000 (0.989, 1.011)
lag4	1.006 (0.996, 1.016)	1.001 (0.995, 1.008)	1.000 (0.978, 1.023)	0.997 (0.975, 1.019)	1.000 (0.999, 1.001)	0.999 (0.988, 1.011)
lag5	**1.010 (1.001, 1.020)**	1.004 (0.998, 1.011)	1.013 (0.991, 1.036)	0.998 (0.977, 1.019)	1.000 (0.999, 1.001)	0.993 (0.982, 1.005)
lag6	**1.011 (1.001, 1.021)**	1.005 (0.998, 1.011)	**1.032 (1.010, 1.055)**	1.008 (0.987, 1.029)	1.001 (1.000, 1.001)	0.999 (0.988, 1.011)
lag7	1.003 (0.993, 1.014)	1.002 (0.995, 1.009)	1.015 (0.993, 1.038)	0.992 (0.971, 1.013)	1.000 (0.999, 1.001)	**1.019 (1.008, 1.031)**
lag01	0.991 (0.978, 1.005)	0.990 (0.980, 0.999)	0.988 (0.957, 1.021)	1.011 (0.983, 1.040)	1.000 (0.999, 1.001)	0.986 (0.969,1.003)
lag02	1.000 (0.985, 1.015)	0.996 (0.985, 1.007)	1.006 (0.966, 1.048)	1.029 (0.995, 1.064)	1.000 (0.999, 1.001)	0.981 (0.964, 0.999)
lag03	1.005 (0.989, 1.021)	1.001 (0.989, 1.012)	1.018 (0.970, 1.069)	1.032 (0.995, 1.071)	1.000 (0.999, 1.002)	0.985 (0.966, 1.004)
lag04	1.009 (0.992, 1.026)	1.002 (0.990, 1.014)	1.019 (0.965, 1.076)	1.028 (0.988, 1.069)	1.000 (0.999, 1.001)	0.986 (0.966, 1.006)
lag05	1.014 (0.997, 1.032)	1.005 (0.993, 1.018)	1.042 (0.983, 1.104)	1.028 (0.986, 1.071)	1.000 (0.999, 1.002)	0.982 (0.961, 1.004)
lag06	**1.019 (1.001, 1.038)**	1.009 (0.995, 1.022)	**1.083 (1.018, 1.152)**	1.033 (0.990, 1.078)	1.001 (0.999, 1.002)	0.982 (0.959, 1.006)
lag07	**1.021 (1.002, 1.041)**	1.010 (0.995, 1.024)	**1.103 (1.032, 1.178)**	1.030 (0.986, 1.077)	1.001 (0.999, 1.002)	0.992 (0.967, 1.019)

Note: statistically significant ($P < 0.05$) are indicated in bold.

Table S3. Relative risk and 95% CI of hospital admissions for anxiety disorders at different lag days for every 10 μg/m^3 increase in pollutant concentration

	$PM_{2.5}$ RR(95%CI)	PM_{10} RR(95%CI)	SO_2 RR(95%CI)	NO_2 RR(95%CI)	CO RR(95%CI)	O_3 RR(95%CI)
lag0	1.001 (0.993, 1.009)	1.000 (0.994, 1.005)	1.004 (0.990, 1.018)	1.004 (0.987, 1.021)	1.000 (0.999, 1.001)	1.001 (0.991, 1.010)
lag1	1.002 (0.994, 1.010)	0.998 (0.993, 1.004)	1.001 (0.987, 1.015)	1.005 (0.988, 1.022)	1.000 (1.000, 1.001)	1.000 (0.991, 1.009)
lag2	1.006 (0.998, 1.013)	1.002 (0.997,1.008)	1.008 (0.993, 1.023)	1.003 (0.986, 1.020)	1.000 (1.000, 1.001)	1.004 (0.996, 1.013)
lag3	1.004 (0.997, 1.012)	1.003 (0.997, 1.008)	1.011 (0.996, 1.025)	0.996 (0.979, 1.013)	1.001 (1.000, 1.001)	1.006 (0.998, 1.013)
lag4	1.007 (1.000, 1.015)	1.003 (0.998, 1.008)	**1.022 (1.007, 1.037)**	1.013 (0.996, 1.031)	**1.001 (1.000, 1.001)**	1.002 (0.994, 1.010)
lag5	1.003 (0.995, 1.011)	1.000 (0.994, 1.005)	1.006 (0.991, 1.021)	1.001 (0.984, 1.017)	1.000 (0.999, 1.000)	1.000 (0.992, 1.008)
lag6	0.999 (0.992, 1.008)	0.998 (0.993, 1.003)	1.003 (0.989, 1.018)	0.991 (0.974, 1.007)	1.000 (0.999, 1.000)	1.001 (0.993, 1.009)
lag7	0.995 (0.987, 1.003)	0.996 (0.991, 1.002)	0.995 (0.980, 1.010)	0.987 (0.971, 1.004)	1.000 (0.999, 1.000)	1.004 (0.995, 1.013)
lag01	1.000 (0.989, 1.011)	0.996 (0.988,1.004)	1.003 (0.980, 1.028)	1.005 (0.982, 1.030)	1.000 (0.999, 1.001)	1.000 (0.986, 1.015)
lag02	1.003 (0.991, 1.016)	0.998 (0.989, 1.007)	1.010 (0.980, 1.041)	1.003 (0.976, 1.031)	1.000 (0.999, 1.001)	1.005 (0.990, 1.020)
lag03	1.005 (0.992, 1.018)	1.000 (0.991, 1.010)	1.018 (0.982, 1.056)	0.998 (0.968, 1.029)	1.001 (1.000, 1.002)	1.009 (0.993, 1.026)
lag04	1.009 (0.995, 1.023)	1.003 (0.993, 1.013)	**1.047 (1.005, 1.091)**	1.008 (0.976, 1.041)	**1.001 (1.000, 1.003)**	1.008 (0.991, 1.025)
lag05	1.009 (0.995, 1.023)	1.002 (0.992, 1.013)	**1.053 (1.008, 1.100)**	1.007 (0.974, 1.042)	1.001 (1.000, 1.002)	1.005 (0.987, 1.023)
lag06	1.008 (0.993, 1.023)	1.001 (0.990,1.012)	**1.058 (1.009, 1.110)**	1.002 (0.967, 1.037)	1.001 (1.000, 1.002)	1.006 (0.986, 1.026)
lag07	1.005 (0.989, 1.021)	0.999 (0.987,1.011)	1.049 (0.997, 1.104)	0.994 (0.959, 1.030)	1.001 (0.999, 1.002)	1.010 (0.989, 1.032)

Note: statistically significant ($P < 0.05$) are indicated in bold.

Table S4. Relative risk and 95% CI of hospital admissions for mental and behavioral disorders at different lag days for every 10 μg/m^3 increase in pollutants after seasonal stratification

	$PM_{2.5}$ RR(95%CI)		PM_{10} RR(95%CI)		SO_2 RR(95%CI)	
	cold season	warm season	cold season	warm season	cold season	warm season
lag0	0.995 (0.987, 1.003)	0.998 (0.985, 1.011)	0.996 (0.990, 1.002)	0.995 (0.988, 1.003)	1.000 (0.986, 1.014)	1.001 (0.970, 1.032)
lag1	0.997 (0.989, 1.004)	0.990 (0.977, 1.003)	0.996 (0.990, 1.002)	0.992 (0.984, 0.999)	0.991 (0.977, 1.005)	0.979 (0.948, 1.011)
lag2	1.002 (0.995, 1.009)	1.007 (0.993, 1.020)	0.999 (0.994, 1.005)	1.006 (0.999, 1.013)	1.000 (0.985, 1.014)	1.025 (0.990, 1.060)
lag3	1.003 (0.996, 1.009)	1.000 (0.987, 1.014)	1.001 (0.996, 1.007)	1.006 (0.999, 1.013)	1.005 (0.991, 1.020)	1.005 (0.974, 1.036)
lag4	1.004 (0.997, 1.011)	1.001 (0.987, 1.014)	1.001 (0.996, 1.006)	1.003 (0.996, 1.010)	1.011 (0.997, 1.025)	1.011 (0.980, 1.044)
lag5	1.004 (0.997, 1.011)	0.995 (0.982, 1.008)	1.000 (0.995, 1.005)	0.998 (0.991, 1.005)	1.005 (0.991, 1.020)	0.996 (0.965, 1.028)
lag6	1.007 (0.993, 1.008)	1.002 (0.989, 1.015)	0.999 (0.993, 1.004)	1.001 (0.994, 1.008)	1.005 (0.992, 1.020)	1.008 (0.975, 1.043)
lag7	0.995 (0.988, 1.003)	1.003 (0.990, 1.017)	0.997 (0.991, 1.003)	1.002 (0.995, 1.010)	0.997 (0.983, 1.011)	1.000 (0.967, 1.034)
lag01	0.999 (0.988, 1.010)	0.992 (0.971, 1.014)	0.998 (0.989, 1.006)	0.986 (0.975, 0.998)	0.998 (0.975, 1.022)	1.001 (0.948, 1.057)
lag02	1.004 (0.992, 1.016)	1.001 (0.977, 1.026)	0.999 (0.990, 1.009)	0.994 (0.981, 1.008)	1.002 (0.972, 1.032)	1.035 (0.968, 1.106)
lag03	1.007 (0.994, 1.020)	1.003 (0.976, 1.030)	1.002 (0.992, 1.012)	1.001 (0.987, 1.017)	1.010 (0.973, 1.048)	1.036 (0.961, 1.117)
lag04	1.011 (0.997, 1.025)	1.003 (0.973,1.034)	1.004 (0.993, 1.014)	1.002 (0.985, 1.020)	1.029 (0.986, 1.073)	1.047 (0.962, 1.140)
lag05	1.014 (0.999, 1.029)	0.999 (0.967, 1.033)	1.004 (0.993, 1.015)	0.998 (0.979, 1.017)	1.043 (0.997, 1.092)	1.053 (0.960, 1.154)
lag06	1.015 (1.000, 1.031)	1.002 (0.967, 1.038)	1.004 (0.993, 1.016)	0.998 (0.978, 1.019)	**1.059 (1.007, 1.114)**	1.066 (0.967, 1.174)
lag07	1.014 (0.997, 1.031)	1.003 (0.965, 1.042)	1.004 (0.991 ,1.016)	0.998 (0.976, 1.020)	**1.058 (1.002, 1.118)**	1.071 (0.967, 1.185)

	NO_2 RR(95%CI)		CO RR(95%CI)		O_3 RR(95%CI)	
	cold season	warm season	cold season	warm season	cold season	warm season
lag0	1.003 (0.984, 1.023)	0.999 (0.980, 1.019)	1.000 (0.999, 1.000)	1.000 (0.999, 1.000)	0.997 (0.982, 1.012)	0.997 (0.988, 1.007)
lag1	0.999 (0.980, 1.018)	0.991 (0.972, 1.011)	1.000 (0.999, 1.000)	1.000 (0.999, 1.000)	0.996 (0.983, 1.010)	1.001 (0.993, 1.009)
lag2	1.008 (0.989, 1.026)	0.999 (0.979, 1.020)	1.000 (1.000, 1.001)	1.000 (1.000, 1.001)	1.000 (0.987, 1.012)	1.002 (0.995, 1.009)
lag3	1.009 (0.992, 1.027)	0.978 (0.958, 0.998)	1.000 (1.000, 1.001)	1.000 (0.999, 1.001)	1.006 (0.994, 1.018)	1.004 (0.998, 1.011)
lag4	1.015 (0.998, 1.033)	0.987 (0.966, 1.008)	1.000 (1.000, 1.001)	1.000 (0.999, 1.001)	0.998 (0.985, 1.012)	1.002 (0.995, 1.009)
lag5	1.006 (0.989, 1.023)	0.977 (0.957, 0.997)	1.000 (1.000, 1.001)	1.000 (0.999, 1.000)	0.996 (0.982, 1.010)	1.000 (0.993, 1.007)
lag6	0.999 (0.983, 1.017)	0.979 (0.958, 0.999)	1.000 (1.000, 1.001)	1.000 (0.999, 1.000)	1.002 (0.990, 1.015)	1.004 (0.997, 1.011)
lag7	0.989 (0.972, 1.006)	0.981 (0.961, 1.002)	1.000 (0.999, 1.000)	1.000 (0.999, 1.001)	**1.012 (1.000, 1.024)**	**1.008 (1.000, 1.015)**
lag01	1.011 (0.983, 1.040)	1.012 (0.982, 1.043)	1.000 (0.999, 1.001)	1.000 (0.999, 1.001)	0.990 (0.966, 1.014)	0.996 (0.982, 1.010)
lag02	1.021 (0.988, 1.055)	1.018 (0.982, 1.054)	1.000 (0.999, 1.002)	1.000 (0.999, 1.002)	0.990 (0.966, 1.015)	0.998 (0.983, 1.012)
lag03	1.029 (0.992, 1.068)	1.003 (0.964, 1.044)	1.001 (1.000, 1.002)	1.000 (0.999, 1.002)	0.997 (0.972, 1.023)	1.001 (0.986, 1.016)
lag04	**1.042 (1.001, 1.085)**	0.999 (0.955, 1.044)	**1.001 (1.000, 1.003)**	1.000 (0.998, 1.002)	0.996 (0.968, 1.024)	1.000 (0.985, 1.016)
lag05	**1.044 (1.002, 1.088)**	0.988 (0.941, 1.038)	1.001 (1.000, 1.003)	1.000 (0.998, 1.002)	0.992 (0.962, 1.022)	0.997 (0.980, 1.014)
lag06	1.042 (0.998, 1.087)	0.978 (0.928, 1.031)	1.001 (1.000, 1.003)	1.000 (0.998, 1.002)	0.992 (0.960, 1.025)	1.000 (0.981, 1.019)
lag07	1.031 (0.986, 1.078)	0.969 (0.917, 1.023)	1.001 (1.000, 1.003)	1.000 (0.997, 1.002)	1.001 (0.968, 1.035)	1.004 (0.984, 1.024)

Note: statistically significant ($P < 0.05$) are indicated in bold. *P value obtained from the Z-test for the difference of the effect estimates of air pollutants between seasonal groups. No differences between groups were found after Z-test.

Table S5. Relative risk and 95% CI of depression hospital admissions at different lag days for every 10 μg/m^3 increase in pollutants after seasonal stratification

	$PM_{2.5}$ RR(95%CI)		PM_{10} RR(95%CI)		SO_2 RR(95%CI)	
	cold season	warm season	cold season	warm season	cold season	warm season
lag0	0.984 (0.970, 0.998)	1.000 (0.979, 1.022)	0.988 (0.978, 0.997)	0.995 (0.983, 1.007)	0.990 (0.964, 1.017)	1.014 (0.963, 1.068)
lag1	1.000 (0.998, 1.013)	0.982 (0.961, 1.004)	0.997 (0.987, 1.0060	0.991 (0.978, 1.004)	0.992 (0.965, 1.020)	0.983 (0.932, 1.037)
lag2	**1.014 (1.003, 1.025)**	0.989 (0.967, 1.011)	1.006 (0.997, 1.0150	1.003 (0.990, 1.015)	1.021 (0.994, 1.047)	1.016 (0.962, 1.073)
lag3	**1.013 (1.002, 1.024)**	0.989 (0.968, 1.010)	1.008 (1.000, 1.016)	1.006 (0.994, 1.018)	1.012 (0.986, 1.039)	0.986 (0.937, 1.037)
lag4	**1.015 (1.003, 1.026)**	0.989 (0.968, 1.010)	1.005 (0.998, 1.013)	0.997 (0.985, 1.009)	1.005 (0.980, 1.030)	0.980 (0.933, 1.031)
lag5	**1.020 (1.009, 1.032)**	0.991 (0.970, 1.011)	**1.011 (1.003, 1.019)**	0.994 (0.982, 1.005)	**1.026 (1.000, 1.051)**	0.973 (0.925, 1.022)
lag6	**1.019 (1.007, 1.031)**	1.005 (0.985, 1.026)	**1.009 (1.001, 1.017)**	1.003 (0.991, 1.015)	**1.031 (1.006, 1.056)**	1.051 (0.996, 1.108)
lag7	1.008 (0.996, 1.020)	1.003 (0.982, 1.024)	1.006 (0.998, 1.014)	1.002 (0.990, 1.015)	1.009 (0.984, 1.035)	1.046 (0.994, 1.101)
lag01	0.985 (0.969, 1.002)	0.997 (0.965, 1.030)	0.986 (0.974, 0.998)	0.992 (0.974, 1.010)	0.974 (0.937, 1.012)	1.022 (0.940, 1.110)
lag02	0.997 (0.980, 1.016)	0.992 (0.956, 1.030)	0.991 (0.978, 1.005)	0.997 (0.977, 1.018)	0.991 (0.944, 1.040)	1.042 (0.942, 1.153)
lag03	1.005 (0.986, 1.024)	0.987 (0.947, 1.028)	0.997 (0.982, 1.011)	1.005 (0.982, 1.029)	1.000 (0.942, 1.062)	1.025 (0.913, 1.151)
lag04	1.011 (0.991, 1.032)	0.979 (0.935, 1.025)	1.000 (0.985, 1.015)	1.004 (0.978, 1.031)	1.002 (0.937, 1.072)	1.007 (0.881, 1.149)
lag05	1.020 (0.998, 1.042)	0.975 (0.929, 1.024)	1.006 (0.990, 1.0220	0.998 (0.970, 1.027)	1.040 (0.967, 1.118)	1.003 (0.869, 1.157)
lag06	**1.028 (1.005, 1.051)**	0.981 (0.932, 1.033)	1.01) (0.994, 1.028)	1.001 (0.971, 1.032)	**1.087 (1.005, 1.175)**	1.077 (0.926, 1.252)
lag07	**1.031 (1.007, 1.056)**	0.978 (0.923, 1.035)	1.014 (0.996, 1.033)	1.001 (0.968, 1.034)	**1.097 (1.008, 1.195)**	1.136 (0.968, 1.333)

	NO_2 RR(95%CI)		CO RR(95%CI)		O_3 RR(95%CI)	
	cold season	warm season	cold season	warm season	cold season	warm season
lag0	0.992 (0.962, 1.023)	1.026 (0.993, 1.060)	0.999 (0.998, 1.000)	1.000 (0.999, 1.001)	1.012 (0.982, 1.043)	0.982 (0.967, 0.998)
lag1	1.005 (0.974, 1.037)	1.001 (0.968, 1.036)	1.000 (0.999, 1.001)	0.999 (0.998, 1.000)	0.993 (0.970, 1.017)	0.994 (0.981, 1.008)
lag2	**1.038 (1.008, 1.069)**	1.002 (0.968, 1.037)	**1.001 (1.000, 1.002)**	0.999 (0.998, 1.001)	0.990 (0.969, 1.012)	0.993 (0.981, 1.006)
lag3	1.019 (0.990, 1.048)	0.985 (0.953, 1.019)	1.001 (1.000, 1.002)	0.999 (0.998, 1.000)	1.017 (0.995, 1.038)	1.000 (0.988, 1.012)
lag4	1.017 (0.989, 1.046)	0.973 (0.939, 1.008)	1.001 (1.000, 1.002)	0.999 (0.998, 1.000)	1.018 (0.995, 1.042)	0.999 (0.987, 1.010)
lag5	1.024 (0.997, 1.052)	0.975 (0.943, 1.009)	**1.001 (1.000, 1.002)**	0.999 (0.998, 1.000)	1.002 (0.979, 1.026)	0.991 (0.979, 1.003)
lag6	1.027 (1.000, 1.055)	0.997 (0.964, 1.032)	**1.001 (1.000, 1.002)**	1.000 (0.999, 1.001)	1.001 (0.980, 1.022)	0.999 (0.986, 1.011)
lag7	1.002 (0.975, 1.030)	0.991 (0.959, 1.025)	1.000 (1.000, 1.001)	1.000 (0.999, 1.001)	1.017 (0.997, 1.037)	**1.014 (1.002, 1.026)**
lag01	0.983 (0.943, 1.024)	1.038 (0.992, 1.086)	0.999 (0.998, 1.001)	1.000 (0.998, 1.002)	1.000 (0.964, 1.036)	0.981 (0.960, 1.002)
lag02	1.010 (0.962, 1.061)	1.042 (0.988, 1.100)	1.001 (0.999, 1.002)	1.000 (0.998, 1.002)	0.990 (0.955, 1.027)	0.983 (0.962, 1.006)
lag03	1.019 (0.965, 1.076)	1.029 (0.969, 1.093)	1.001 (0.999, 1.003)	0.999 (0.997, 1.002)	1.003 (0.966, 1.041)	0.988 (0.966, 1.011)
lag04	1.023 (0.965, 1.085)	1.010 (0.944, 1.081)	1.001 (0.999, 1.003)	0.998 (0.995, 1.000)	1.014 (0.974, 1.055)	0.990 (0.966, 1.014)
lag05	1.036 (0.975, 1.101)	1.004 (0.932, 1.081)	1.002 (1.000, 1.004)	0.998 (0.995, 1.000)	1.016 (0.973, 1.060)	0.983 (0.958, 1.010)
lag06	1.052 (0.988, 1.120)	1.008 (0.931, 1.091)	**1.002 (1.000, 1.004)**	0.998 (0.995, 1.001)	1.015 (0.970, 1.063)	0.985 (0.956, 1.013)
lag07	1.046 (0.980, 1.117)	1.004 (0.923, 1.091)	**1.003 (1.001, 1.005)**	0.998 (0.995, 1.001)	1.026 (0.979, 1.075)	0.990 (0.960, 1.022)

Note: statistically significant ($P < 0.05$) are indicated in bold.*P value obtained from the Z-test for the difference of the effect estimates of air pollutants between seasonal groups. No differences between groups were found after Z-test.

Table S6. Relative risk and 95% CI of hospital admissions for anxiety disorders at different lag days for every 10 μg/m^3 increase in pollutants after seasonal stratification

	$PM_{2.5}$ RR(95%CI)		PM_{10} RR(95%CI)		SO_2 RR(95%CI)	
	cold season	warm season	cold season	warm season	cold season	warm season
lag0	1.002<br>(0.991, 1.012)	0.997<br>(0.979, 1.014)	1.001<br>(0.993, 1.008)	0.996<br>(0.986, 1.006)	1.002<br>(0.985, 1.020)	1.010<br>(0.969, 1.054)
lag1	1.004<br>(0.995, 1.014)	0.993<br>(0.975, 1.010)	1.001<br>(0.994, 1.009)	0.991<br>(0.981, 1.001)	1.001<br>(0.984, 1.018)	0.986<br>(0.944, 1.031)
lag2	1.005<br>(0.996, 1.013)	1.011<br>(0.993, 1.029)	1.001<br>(0.994, 1.008)	1.005<br>(0.995, 1.014)	1.006<br>(0.988, 1.024)	1.037<br>(0.991, 1.086)
lag3	1.005<br>(0.997, 1.013)	1.002<br>(0.984, 1.020)	1.002<br>(0.996, 1.009)	1.003<br>(0.994, 1.012)	1.011<br>(0.994, 1.029)	1.029<br>(0.986, 1.074)
lag4	1.005<br>(0.996, 1.013)	1.016<br>(0.998, 1.034)	1.000<br>(0.994, 1.007)	1.008<br>(0.999, 1.017)	**1.021**<br>**(1.003, 1.039)**	**1.064**<br>**(1.018, 1.112)**
lag5	1.001<br>(0.993, 1.010)	1.001<br>(0.984, 1.019)	0.997<br>(0.990, 1.004)	1.000<br>(0.991, 1.009)	1.001<br>(0.983, 1.020)	1.036<br>(0.993, 1.081)
lag6	0.997<br>(0.988, 1.007)	1.002<br>(0.985, 1.020)	0.996<br>(0.989, 1.003)	1.000<br>(0.990, 1.009)	1.003<br>(0.986, 1.020)	1.003<br>(0.958, 1.051)
lag7	0.992<br>(0.982, 1.002)	1.003<br>(0.984, 1.021)	0.994<br>(0.987, 1.001)	1.000<br>(0.990, 1.010)	0.994<br>(0.977, 1.012)	0.975<br>(0.931, 1.021)
lag01	1.008,<br>(0.994, 1.022)	0.986<br>(0.959, 1.014)	1.003<br>(0.993, 1.014)	0.987<br>(0.971, 1.002)	1.005<br>(0.977, 1.034)	0.983<br>(0.914, 1.057)
lag02	1.011<br>(0.996, 1.026)	0.998<br>(0.966, 1.030)	1.004<br>(0.993, 1.016)	0.993<br>(0.976, 1.011)	1.013<br>(0.977, 1.050)	1.017<br>(0.932, 1.110)
lag03	1.014<br>(0.998, 1.031)	0.999<br>(0.965, 1.035)	1.006<br>(0.994, 1.019)	0.996<br>(0.977, 1.015)	1.025<br>(0.980, 1.072)	1.046<br>(0.947, 1.154)
lag04	**1.018**<br>**(1.000, 1.036)**	1.010<br>(0.971, 1.051)	1.007<br>(0.994, 1.020)	1.001<br>(0.979, 1.023)	**1.060**<br>**(1.007, 1.115)**	1.105<br>(0.990, 1.234)
lag05	1.018<br>(1.000, 1.037)	1.008<br>(0.966, 1.052)	1.005<br>(0.991, 1.019)	1.000<br>(0.976, 1.025)	**1.068**<br>**(1.010, 1.130)**	**1.126**<br>**(1.000, 1.268)**
lag06	1.017<br>(0.997, 1.037)	1.010<br>(0.964, 1.057)	1.003<br>(0.988, 1.018)	1.000<br>(0.974, 1.027)	**1.078**<br>**(1.012, 1.147)**	1.116<br>(0.985, 1.265)
lag07	1.013<br>(0.992, 1.034)	1.008<br>(0.958, 1.060)	1.001<br>(0.985, 1.017)	0.997<br>(0.969, 1.027)	**1.071**<br>**(1.000, 1.147)**	1.081<br>(0.948, 1.234)

	NO_2 RR(95%CI)		CO RR(95%CI)		O_3 RR(95%CI)	
	cold season	warm season	cold season	warm season	cold season	warm season
lag0	1.011 (0.986, 1.036)	0.993 (0.967, 1.020)	1.000 (0.999, 1.001)	1.000 (0.999, 1.001)	0.998 (0.979, 1.017)	1.001 (0.988, 1.014)
lag1	1.010 (0.986, 1.034)	0.992 (0.965, 1.020)	1.000 (1.000, 1.001)	1.000 (0.999, 1.001)	1.001 (0.984, 1.017)	1.002 (0.991, 1.012)
lag2	1.008 (0.984, 1.031)	0.993 (0.966, 1.022)	1.000 (1.000, 1.001)	1.001 (1.000, 1.002)	1.003 (0.988, 1.019)	1.006 (0.996, 1.015)
lag3	1.016 (0.994, 1.039)	0.969 (0.942, 0.997)	1.001 (1.000, 1.001)	1.000 (1.000, 1.001)	1.002 (0.986, 1.017)	1.006 (0.997, 1.015)
lag4	1.022 (0.999, 1.045)	1.008 (0.978, 1.039)	1.001 (1.000, 1.001)	1.001 (1.000, 1.002)	0.988 (0.971, 1.005)	1.002 (0.993, 1.011)
lag5	1.001 (0.980, 1.024)	0.999 (0.971, 1.027)	1.000 (0.999, 1.000)	1.000 (0.999, 1.001)	0.992 (0.974, 1.010)	1.000 (0.991, 1.010)
lag6	0.988 (0.967, 1.009)	0.989 (0.960, 1.018)	1.000 (0.999, 1.000)	0.999 (0.998, 1.000)	1.002 (0.987, 1.018)	0.999 (0.990, 1.009)
lag7	0.983 (0.962, 1.005)	0.985 (0.957, 1.014)	1.000 (0.999, 1.000)	1.000 (0.999, 1.001)	1.011 (0.996, 1.026)	1.000 (0.990, 1.010)
lag01	1.022 (0.986, 1.058)	0.992 (0.954, 1.033)	1.001 (0.999, 1.002)	0.999 (0.998, 1.001)	0.996 (0.966, 1.027)	1.004 (0.985, 1.022)
lag02	1.027 (0.986, 1.071)	0.988 (0.943, 1.035)	1.001 (1.000, 1.002)	1.000 (0.998, 1.002)	1.000 (0.969, 1.032)	1.009 (0.991, 1.028)
lag03	1.038 (0.991, 1.087)	0.968 (0.919, 1.020)	**1.002 (1.000, 1.003)**	1.000 (0.998, 1.002)	1.003 (0.970, 1.036)	1.013 (0.994, 1.033)
lag04	**1.058 (1.006, 1.113)**	0.977 (0.922, 1.036)	**1.002 (1.001, 1.004)**	1.001 (0.999, 1.004)	0.993 (0.958, 1.030)	1.011 (0.991, 1.032)
lag05	**1.055 (1.001, 1.112)**	0.974 (0.913, 1.039)	1.002 (1.000, 1.003)	1.001 (0.999, 1.004)	0.988 (0.949, 1.027)	1.007 (0.986, 1.029)
lag06	1.040 (0.985, 1.098)	0.967 (0.903, 1.036)	1.001 (1.000, 1.003)	1.000 (0.998, 1.003)	0.991 (0.950, 1.034)	1.008 (0.984, 1.032)
lag07	1.026 (0.969, 1.086)	0.954 (0.888, 1.025)	1.001 (0.999, 1.003)	1.000 (0.997, 1.003)	1.001 (0.959, 1.046)	1.009 (0.983, 1.035)

Note: statistically significant ($P < 0.05$) are indicated in bold. *P value obtained from the Z-test for the difference of the effect estimates of air pollutants between seasonal groups. No differences between groups were found after Z-test.

Table S7. Relative risk and 95% CI of hospital admissions for mental and behavioral disorders at different lag days for every 10 μg/m^3 increase in pollutants after age stratification

	$PM_{2.5}$ RR(95%CI)			PM_{10} RR(95%CI)		
	<18	18-60	>60	<18	18-60	>60
lag0	1.005 (0.993, 1.017)	0.996 (0.989, 1.003)	0.999 (0.988, 1.011)	1.002 (0.995, 1.010)	0.995 (0.990, 0.999)	1.000 (0.992, 1.008)
lag1	0.995 (0.984, 1.006)	0.998 (0.991, 1.005)	0.995 (0.984, 1.007)	0.999 (0.991, 1.007)	0.996 (0.991, 1.001)	0.994 (0.986, 1.003)
lag2	1.007 (0.997, 1.018)	1.002 (0.996, 1.009)	1.008 (0.998, 1.018)	**1.008 (1.001, 1.015)**	1.000 (0.996, 1.005)	1.005 (0.997, 1.013)
lag3	1.004 (0.994, 1.014)	1.003 (0.997, 1.009)	0.995 (0.985, 1.006)	1.004 (0.997, 1.011)	1.003 (0.998, 1.007)	0.999 (0.991, 1.007)
lag4	1.000 (0.990, 1.011)	1.004 (0.998, 1.011)	1.001 (0.990, 1.012)	1.000 (0.993, 1.007)	1.002 (0.997, 1.006)	0.999 (0.992, 1.006)
lag5	0.996 (0.985, 1.006)	1.006 (0.999, 1.013)	0.996 (0.986, 1.007)	0.996 (0.989, 1.003)	1.002 (0.997, 1.006)	0.995 (0.987, 1.002)
lag6	0.997 (0.987, 1.008)	1.003 (0.996, 1.010)	0.998 (0.987, 1.010)	0.996 (0.989, 1.003)	1.001 (0.996, 1.006)	0.998 (0.990, 1.005)
lag7	1.004 (0.993, 1.015)	0.996 (0.989, 1.003)	1.001 (0.989, 1.012)	1.003 (0.996, 1.011)	0.996 (0.992, 1.001)	0.999 (0.991, 1.007)
lag01	1.002 (0.988, 1.016)	0.998 (0.988, 1.007)	0.987 (0.972, 1.003)	1.002 (0.992, 1.012)	0.993 (0.987, 1.000)	0.990 (0.979, 1.002)
lag02	1.007 (0.992, 1.023)	1.000 (0.990, 1.010)	0.995 (0.978, 1.012)	1.007 (0.996, 1.018)	0.995 (0.988, 1.003)	0.996 (0.984, 1.009)
lag03	1.009 (0.993, 1.026)	1.003 (0.992, 1.014)	0.991 (0.973, 1.009)	1.008 (0.996, 1.021)	0.999 (0.991, 1.007)	0.995 (0.982, 1.009)
lag04	1.010 (0.992, 1.027)	1.005 (0.994, 1.017)	0.992 (0.973, 1.011)	1.007 (0.995, 1.020)	1.001 (0.992, 1.009)	0.995 (0.981, 1.010)
lag05	1.006 (0.988, 1.025)	1.009 (0.997, 1.021)	0.990 (0.970, 1.010)	1.003 (0.990, 1.017)	1.003 (0.994, 1.011)	0.993 (0.978, 1.008)
lag06	1.006 (0.987, 1.026)	1.010 (0.998, 1.023)	0.989 (0.968, 1.010)	1.001 (0.988, 1.016)	1.004 (0.994, 1.013)	0.991 (0.976, 1.007)
lag07	1.008 (0.988, 1.029)	1.009 (0.995, 1.022)	0.988 (0.967, 1.010)	1.003 (0.988, 1.018)	1.002 (0.992, 1.012)	0.990 (0.974, 1.007)

	SO2 RR(95%CI)			NO2 RR(95%CI)		
	<18	18-60	>60	<18	18-60	>60
lag0	1.010 (0.985, 1.035)	0.999 (0.986, 1.013)	1.002 (0.981, 1.024)	1.014 (0.992, 1.037)	0.998 (0.984, 1.013)	1.006 (0.982, 1.031)
lag1	0.976 (0.951, 1.001)	0.996 (0.983, 1.010)	1.001 (0.979, 1.023)	0.993 (0.970, 1.017)	1.001 (0.986, 1.016)	1.003 (0.979, 1.028)
lag2	1.003 (0.978, 1.029)	1.001 (0.987, 1.015)	**1.033 (1.011, 1.056)**	1.008 (0.985, 1.032)	1.002 (0.987, 1.017)	1.022 (0.997, 1.047)
lag3	1.019 (0.994, 1.044)	1.004 (0.990, 1.017)	1.007 (0.985, 1.029)	1.014 (0.991, 1.037)	0.992 (0.978, 1.006)	1.001 (0.977, 1.026)
lag4	1.012 (0.988, 1.036)	1.010 (0.996, 1.024)	1.013 (0.991, 1.035)	1.000 (0.977, 1.023)	1.002 (0.988, 1.017)	1.002 (0.978, 1.028)
lag5	0.991 (0.968, 1.016)	1.007 (0.993, 1.021)	1.013 (0.990, 1.037)	0.990 (0.968, 1.012)	0.996 (0.982, 1.010)	1.002 (0.978, 1.027)
lag6	0.990 (0.966, 1.014)	1.007 (0.994, 1.021)	1.007 (0.985, 1.029)	0.983 (0.962, 1.004)	0.990 (0.976, 1.004)	1.002 (0.978, 1.027)
lag7	1.014 (0.989, 1.039)	0.996 (0.982, 1.010)	1.007 (0.985, 1.029)	0.998 (0.977, 1.020)	0.980 (0.966, 0.994)	1.003 (0.979, 1.028)
lag01	0.997 (0.962, 1.034)	1.003 (0.983, 1.024)	0.986 (0.955, 1.018)	1.009 (0.980, 1.039)	1.010 (0.991, 1.031)	0.994 (0.961, 1.028)
lag02	1.005 (0.961, 1.051)	1.004 (0.979, 1.030)	1.012 (0.971, 1.053)	1.015 (0.981, 1.051)	1.012 (0.989, 1.035)	1.007 (0.968, 1.048)
lag03	1.028 (0.975, 1.084)	1.009 (0.978, 1.041)	1.008 (0.959, 1.060)	1.025 (0.986, 1.065)	1.008 (0.982, 1.034)	1.002 (0.959, 1.047)
lag04	1.037 (0.978, 1.100)	1.023 (0.988, 1.060)	1.016 (0.960, 1.074)	1.023 (0.981, 1.066)	1.011 (0.984, 1.039)	1.000 (0.954, 1.047)
lag05	1.030 (0.967, 1.097)	1.033 (0.995, 1.072)	1.033 (0.972, 1.098)	1.012 (0.970, 1.057)	1.012 (0.984, 1.041)	1.001 (0.953, 1.050)
lag06	1.030 (0.963, 1.101)	**1.049 (1.008, 1.092)**	1.032 (0.965, 1.103)	1.006 (0.962, 1.052)	1.010 (0.981, 1.040)	0.997 (0.949, 1.048)
lag07	1.048 (0.977, 1.125)	**1.048 (1.004, 1.094)**	1.030 (0.959, 1.105)	1.006 (0.960, 1.054)	1.003 (0.973, 1.034)	0.996 (0.945, 1.048)

	CO RR(95%CI)			O_3 RR(95%CI)		
	<18	18-60	>60	<18	18-60	>60
lag0	1.000	1.000	1.000	0.987	0.996	0.994
	(0.999, 1.001)	(0.999, 1.000)	(0.999, 1.001)	(0.973, 1.002)	(0.987, 1.005)	(0.979, 1.010)
lag1	1.000	1.000	1.000	1.003	0.995	0.993
	(0.999, 1.000)	(1.000, 1.001)	(0.999, 1.001)	(0.990, 1.015)	(0.987, 1.003)	(0.979, 1.007)
lag2	1.001	1.000	1.001	1.005	0.998	0.993
	(1.000, 1.001)	(1.000, 1.001)	(1.000, 1.001)	(0.993, 1.017)	(0.990, 1.005)	(0.980, 1.006)
lag3	1.000	1.000	1.000	0.999	1.006	0.998
	(1.000, 1.001)	(1.000, 1.001)	(0.999, 1.001)	(0.987, 1.010)	(0.999, 1.013)	(0.986, 1.011)
lag4	1.000	1.000	1.000	0.994	1.003	0.999
	(0.999, 1.001)	(1.000, 1.001)	(0.999, 1.001)	(0.983, 1.006)	(0.996, 1.010)	(0.987, 1.012)
lag5	1.000	1.000	1.000	0.989	1.000	1.001
	(0.999, 1.000)	(1.000, 1.001)	(0.999, 1.000)	(0.977, 1.002)	(0.992, 1.007)	(0.989, 1.014)
lag6	1.000	1.000	1.000	1.002	1.002	0.996
	(0.999, 1.000)	(1.000, 1.001)	(0.999, 1.001)	(0.991, 1.015)	(0.995, 1.010)	(0.984, 1.009)
lag7	1.000	1.000	1.000	1.009	**1.011**	1.003
	(0.999, 1.000)	(0.999, 1.000)	(0.999, 1.001)	(0.997, 1.021)	**(1.003, 1.018)**	(0.990, 1.017)
lag01	1.000	1.000	0.999	0.994	0.994	0.984
	(0.999, 1.001)	(0.999, 1.001)	(0.998, 1.001	(0.976, 1.012)	(0.982, 1.006)	(0.963, 1.005)
lag02	1.001	1.000	1.000	0.995	0.995	0.983
	(0.999, 1.002)	(0.999, 1.001)	(0.999, 1.001)	(0.977, 1.015)	(0.983, 1.007)	(0.961, 1.005)
lag03	1.001	1.000	1.000	0.993	0.999	0.986
	(1.000, 1.002)	(0.999, 1.001)	(0.998, 1.001)	(0.973, 1.013)	(0.986, 1.012)	(0.963, 1.009)
lag04	1.001	1.000	1.000	0.989	0.999	0.987
	(0.999, 1.002)	(0.999, 1.001)	(0.998, 1.001)	(0.968, 1.010)	(0.986, 1.013)	(0.963, 1.012)
lag05	1.000	1.001	0.999	0.979	0.997	0.989
	(0.999, 1.002)	(1.000, 1.002)	(0.998, 1.001)	(0.957, 1.002)	(0.983, 1.012)	(0.963, 1.016)
lag06	1.000	1.001	0.999	0.978	0.999	0.989
	(0.999, 1.002)	(1.000, 1.002)	(0.998, 1.001)	(0.954, 1.004)	(0.983, 1.015)	(0.961, 1.019)
lag07	1.000	1.001	0.999	0.984	1.004	0.991
	(0.999, 1.002)	(0.999, 1.002)	(0.997, 1.001)	(0.957, 1.011)	(0.987, 1.022)	(0.961, 1.023)

Note: statistically significant ($P < 0.05$) are indicated in bold. *P value obtained from the Z-test for the difference of the effect estimates of air pollutants between age groups. No differences between groups were found after Z-test.

Table S8. Relative risk and 95% CI of depression hospital admissions at different lag days for every 10 μg/m³ increase in pollutants after age stratification

	$PM_{2.5}$ RR(95%CI)			PM_{10} RR(95%CI)		
	<18	18-60	>60	<18	18-60	>60
lag0	0.991 (0.967, 1.016)	0.991 (0.978, 1.003)	0.991 (0.965, 1.017)	1.001 (0.986, 1.017)	0.989 (0.981, 0.998)	0.990 (0.973, 1.008)
lag1	0.994 (0.971, 1.017)	0.997 (0.985, 1.009)	0.994 (0.969, 1.019)	1.002 (0.986, 1.019)	0.996 (0.987, 1.005)	0.986 (0.968, 1.004)
lag2	1.016 (0.995, 1.037)	1.005 (0.994, 1.016)	1.015 (0.992, 1.039)	1.012 (0.997, 1.027)	1.003 (0.995, 1.011)	1.008 (0.991, 1.025)
lag3	1.018 (0.998, 1.039)	1.003 (0.993, 1.014)	1.002 (0.979, 1.026)	1.010 (0.996, 1.025)	1.005 (0.997, 1.013)	1.005 (0.988, 1.022)
lag4	1.010 (0.989, 1.032)	1.004 (0.994, 1.015)	1.004 (0.981, 1.029)	1.007 (0.994, 1.020)	1.001 (0.994, 1.008)	0.990 (0.974, 1.007)
lag5	0.999 (0.977, 1.021)	**1.014 (1.003, 1.024)**	0.994 (0.969, 1.019)	0.999 (0.985, 1.013)	**1.008 (1.001, 1.015)**	0.985 (0.968, 1.002)
lag6	0.995 (0.973, 1.018)	**1.015 (1.004, 1.026)**	0.998 (0.973, 1.024)	0.997 (0.982, 1.011)	1.006 (0.999, 1.014)	0.998 (0.981, 1.015)
lag7	1.014 (0.993, 1.035)	1.003 (0.992, 1.015)	0.984 (0.958, 1.011)	1.006 (0.992, 1.020)	1.000 (0.993, 1.008)	1.001 (0.984, 1.019)
lag01	0.996 (0.967, 1.025)	0.992 (0.977, 1.008)	0.988 (0.952, 1.024)	1.007 (0.988, 1.028)	0.988 (0.977, 0.999)	0.981 (0.955, 1.007)
lag02	1.011 (0.981, 1.043)	0.998 (0.981, 1.015)	1.006 (0.968, 1.046)	1.016 (0.994, 1.039)	0.993 (0.981, 1.005)	0.995 (0.966, 1.024)
lag03	1.022 (0.989, 1.055)	1.001 (0.984, 1.019)	1.008 (0.968, 1.051)	1.020 (0.997, 1.044)	0.998 (0.985, 1.011)	1.001 (0.971, 1.032)
lag04	1.026 (0.992, 1.062)	1.004 (0.986, 1.023)	1.012 (0.970, 1.057)	1.023 (0.998, 1.048)	0.999 (0.986, 1.013)	0.997 (0.966, 1.030)
lag05	1.027 (0.991, 1.065)	1.012 (0.992, 1.031)	1.009 (0.964, 1.056)	1.022 (0.997, 1.049)	1.005 (0.991, 1.019)	0.990 (0.957, 1.025)
lag06	1.030 (0.992, 1.069)	1.018 (0.998, 1.039)	1.009 (0.962, 1.058)	1.024 (0.997, 1.052)	1.008 (0.993, 1.023)	0.992 (0.957, 1.028)
lag07	**1.040 (1.001, 1.082)**	1.020 (0.998, 1.041)	1.003 (0.954, 1.055)	**1.031 (1.002, 1.061)**	1.008 (0.992, 1.024)	0.994 (0.957, 1.033)

	SO_2 RR(95%CI)			NO_2 RR(95%CI)		
	<18	18-60	>60	<18	18-60	>60
lag0	0.993 (0.937, 1.053)	0.999 (0.975, 1.024)	1.000 (0.952, 1.050)	1.009 (0.964, 1.057)	1.002 (0.978, 1.027)	0.987 (0.935, 1.041)
lag1	0.956 (0.898, 1.017)	1.000 (0.974, 1.027)	1.004 (0.954, 1.056)	0.985 (0.938, 1.035)	1.010 (0.984, 1.036)	0.965 (0.913, 1.019)
lag2	1.013 (0.956, 1.074)	1.016 (0.991, 1.042)	**1.061 (1.010, 1.116)**	1.018 (0.969, 1.068)	1.021 (0.996, 1.046)	1.013 (0.959, 1.071)
lag3	1.031 (0.974, 1.092)	1.003 (0.979, 1.028)	1.024 (0.974, 1.076)	1.016 (0.970, 1.064)	1.003 (0.980, 1.027)	0.979 (0.927, 1.033)
lag4	1.035 (0.981, 1.092)	0.993 (0.969, 1.017)	1.017 (0.967, 1.069)	0.998 (0.953, 1.046)	0.995 (0.971, 1.019)	0.978 (0.927, 1.033)
lag5	1.012 (0.960, 1.067)	1.015 (0.991, 1.040)	1.004 (0.954, 1.058)	0.999 (0.954, 1.045)	1.000 (0.977, 1.024)	0.965 (0.915, 1.019)
lag6	1.017 (0.962, 1.074)	**1.032 (1.008, 1.057)**	1.028 (0.978, 1.080)	0.983 (0.940, 1.028)	1.010 (0.988, 1.034)	0.986 (0.936, 1.040)
lag7	1.043 (0.987, 1.103)	1.019 (0.994, 1.044)	0.976 (0.926, 1.029)	1.017 (0.973, 1.063)	0.990 (0.967, 1.013)	0.937 (0.887, 0.989)
lag01	0.937 (0.860, 1.020)	0.997 (0.962, 1.033)	0.978 (0.905, 1.057)	1.003 (0.944, 1.065)	1.016 (0.984, 1.048)	0.983 (0.910, 1.062)
lag02	0.950 (0.856, 1.054)	1.010 (0.967, 1.056)	1.037 (0.941, 1.143)	1.028 (0.958, 1.104)	1.031 (0.994, 1.070)	1.006 (0.920, 1.101)
lag03	0.966 (0.855, 1.091)	1.019 (0.966, 1.075)	1.068 (0.948, 1.204)	1.038 (0.960, 1.123)	1.035 (0.993, 1.078)	1.000 (0.906, 1.104)
lag04	0.995 (0.872, 1.135)	1.012 (0.954, 1.075)	1.080 (0.946, 1.234)	1.033 (0.950, 1.124)	1.029 (0.985, 1.075)	0.993 (0.894, 1.104)
lag05	1.005 (0.874, 1.156)	1.038 (0.973, 1.106)	1.090 (0.944, 1.259)	1.038 (0.950, 1.133)	1.031 (0.985, 1.079)	0.983 (0.880, 1.098)
lag06	1.043 (0.900, 1.208)	**1.079 (1.008, 1.155)**	1.118 (0.958, 1.305)	1.047 (0.956, 1.147)	1.036 (0.988, 1.086)	0.985 (0.880, 1.103)
lag07	1.094 (0.937, 1.277)	**1.101 (1.024, 1.184)**	1.078 (0.915, 1.270)	1.071 (0.975, 1.177)	1.031 (0.981, 1.083)	0.962 (0.855, 1.082)

	CO RR(95%CI)			O_3 RR(95%CI)		
	<18	18-60	>60	<18	18-60	>60
lag0	1.000 (0.999, 1.002)	0.999 (0.999, 1.000)	1.000 (0.998, 1.002)	1.005 (0.973, 1.038)	0.989 (0.973, 1.005)	0.999 (0.966, 1.032)
lag1	0.999 (0.998, 1.001)	1.000 (0.999, 1.001)	1.000 (0.998, 1.002)	1.000 (0.973, 1.027)	0.989 (0.976, 1.002)	1.001 (0.972, 1.031)
lag2	1.001 (1.000, 1.002)	1.000 (1.000, 1.001)	1.001 (1.000, 1.003)	0.990 (0.965, 1.015)	0.988 (0.975, 1.000)	0.993 (0.966, 1.021)
lag3	1.000 (0.999, 1.002)	1.000 (0.999, 1.001)	1.000 (0.998, 1.002)	0.997 (0.973, 1.023)	1.002 (0.990, 1.014)	0.999 (0.974, 1.026)
lag4	1.000 (0.999, 1.002)	1.000 (0.999, 1.000)	1.000 (0.998, 1.002)	0.994 (0.969, 1.019)	1.002 (0.990, 1.015)	0.999 (0.973, 1.026)
lag5	1.000 (0.998, 1.001)	1.000 (1.000, 1.001)	0.999 (0.997, 1.001)	0.989 (0.963, 1.015)	0.991 (0.979, 1.004)	1.016 (0.990, 1.043)
lag6	1.000 (0.998, 1.001)	1.001 (1.000, 1.001)	1.000 (0.999, 1.002)	1.014 (0.988, 1.040)	0.995 (0.983, 1.008)	1.010 (0.983, 1.037)
lag7	1.001 (0.999, 1.002)	1.000 (0.999, 1.001)	0.999 (0.997, 1.001)	1.016 (0.992, 1.042)	**1.019 (1.006, 1.032)**	1.027 (0.999, 1.055)
lag01	1.000 (0.998, 1.002)	0.999 (0.998, 1.001)	1.000 (0.997, 1.003)	0.993 (0.956, 1.031)	0.986 (0.967, 1.004)	0.979 (0.935, 1.026)
lag02	1.001 (0.999, 1.004)	1.000 (0.998, 1.001)	1.002 (0.999, 1.005)	0.974 (0.937, 1.014)	0.984 (0.964, 1.003)	0.979 (0.933, 1.028)
lag03	1.002 (0.999, 1.004)	1.000 (0.998, 1.001)	1.002 (0.998, 1.005)	0.966 (0.926, 1.009)	0.989 (0.968, 1.010)	0.984 (0.935, 1.035)
lag04	1.001 (0.999, 1.004)	1.000 (0.998, 1.001)	1.002 (0.998, 1.005)	0.960 (0.916, 1.005)	0.992 (0.970, 1.014)	0.987 (0.935, 1.042)
lag05	1.001 (0.998, 1.004)	1.000 (0.998, 1.002)	1.001 (0.997, 1.005)	0.945 (0.898, 0.995)	0.986 (0.963, 1.010)	1.005 (0.949, 1.065)
lag06	1.002 (0.999, 1.005)	1.000 (0.999, 1.002)	1.001 (0.997, 1.005)	0.942 (0.890, 0.997)	0.985 (0.960, 1.012)	1.017 (0.955, 1.083)
lag07	1.003 (0.999, 1.006)	1.001 (0.999, 1.002)	1.000 (0.996, 1.004)	0.949 (0.893, 1.010)	0.994 (0.966, 1.023)	1.034 (0.966, 1.106)

Note: statistically significant ($P < 0.05$) are indicated in bold. *P value obtained from the Z-test for the difference of the effect estimates of air pollutants between age groups. No differences between groups were found after Z-test.

Table S9. Relative risk and 95% CI of hospital admissions for anxiety disorders at different lag days for every 10 μg/m^3 increase in pollutants after age stratification

	PM$_{2.5}$ RR(95%CI)			PM$_{10}$ RR(95%CI)		
	<18	18-60	>60	<18	18-60	>60
lag0	1.002 (0.983, 1.022)	0.999 (0.990, 1.008)	1.007 (0.992, 1.022)	1.000 (0.988, 1.013)	0.997 (0.991, 1.003)	1.007 (0.997, 1.017)
lag1	0.992 (0.973, 1.012)	1.004 (0.996, 1.013)	1.000 (0.986, 1.015)	0.993 (0.979, 1.007)	0.999 (0.993, 1.005)	1.000 (0.990, 1.010)
lag2	1.008 (0.990, 1.026)	1.005 (0.997, 1.013)	1.006 (0.993, 1.020)	1.009 (0.996, 1.021)	1.001 (0.995, 1.007)	1.003 (0.993, 1.013)
lag3	1.012 (0.995, 1.030)	1.006 (0.998, 1.014)	0.989 (0.975, 1.002)	1.010 (0.998, 1.023)	1.003 (0.997, 1.008)	0.994 (0.984, 1.004)
lag4	1.011 (0.993, 1.029)	1.008 (1.000, 1.017)	0.996 (0.982, 1.010)	1.005 (0.994, 1.017)	1.003 (0.998, 1.009)	0.998 (0.989, 1.007)
lag5	0.994 (0.976, 1.013)	1.007 (0.998, 1.015)	0.990 (0.977, 1.004)	0.998 (0.985, 1.010)	1.001 (0.995, 1.007)	0.993 (0.983, 1.002)
lag6	0.999 (0.980, 1.017)	1.000 (0.991, 1.009)	0.993 (0.979, 1.007)	0.998 (0.986, 1.011)	0.998 (0.992, 1.004)	0.993 (0.983, 1.003)
lag7	1.007 (0.988, 1.027)	0.992 (0.983, 1.001)	0.997 (0.982, 1.012)	1.007 (0.995, 1.020)	0.994 (0.988, 1.000)	0.994 (0.984, 1.005)
lag01	0.998 (0.973, 1.023)	1.002 (0.989, 1.014)	0.994 (0.974, 1.013)	0.995 (0.977, 1.012)	0.997 (0.988, 1.006)	0.999 (0.984, 1.013)
lag02	1.005 (0.978, 1.033)	1.004 (0.990, 1.017)	0.996 (0.976, 1.018)	1.002 (0.983, 1.022)	0.998 (0.988, 1.007)	0.999 (0.983, 1.015)
lag03	1.014 (0.986, 1.044)	1.006 (0.992, 1.021)	0.987 (0.965, 1.009)	1.009 (0.989, 1.030)	1.000 (0.990, 1.010)	0.993 (0.977, 1.010)
lag04	1.020 (0.989, 1.051)	1.010 (0.995, 1.025)	0.985 (0.961, 1.009)	1.011 (0.990, 1.033)	1.003 (0.992, 1.014)	0.993 (0.975, 1.011)
lag05	1.014 (0.983, 1.047)	1.013 (0.997, 1.029)	0.979 (0.955, 1.005)	1.008 (0.985, 1.031)	1.004 (0.993, 1.015)	0.988 (0.970, 1.007)
lag06	1.013 (0.980, 1.046)	1.013 (0.996, 1.029)	0.976 (0.950, 1.003)	1.006 (0.982, 1.030)	1.004 (0.992, 1.016)	0.984 (0.964, 1.004)
lag07	1.016 (0.981, 1.052)	1.008 (0.991, 1.025)	0.974 (0.947, 1.002)	1.009 (0.984, 1.036)	1.001 (0.988, 1.014)	0.980 (0.959, 1.001)

	SO_2 RR(95%CI)			NO_2 RR(95%CI)		
	<18	18-60	>60	<18	18-60	>60
lag0	1.007 (0.965, 1.050	1.001 (0.986, 1.016)	1.014 (0.988, 1.041)	1.018 (0.979, 1.058)	0.995 (0.976, 1.013)	1.008 (0.977, 1.040)
lag1	0.965 (0.923, 1.009)	1.003 (0.989, 1.018)	1.013 (0.986, 1.040)	0.986 (0.946, 1.028)	1.001 (0.983, 1.020)	1.013 (0.982, 1.045)
lag2	0.993 (0.950, 1.039)	1.006 (0.990, 1.022)	**1.037 (1.009, 1.066)**	0.999 (0.960, 1.041)	0.994 (0.976, 1.013)	1.029 (0.997, 1.061)
lag3	1.027 (0.984, 1.072)	1.010 (0.995, 1.026)	1.002 (0.975, 1.030)	1.021 (0.982, 1.062)	0.991 (0.973, 1.009)	0.991 (0.961, 1.023)
lag4	1.035 (0.993, 1.079)	**1.023 (1.008, 1.039)**	1.010 (0.983, 1.039)	1.011 (0.972, 1.052)	1.013 (0.994, 1.032)	0.992 (0.961, 1.024)
lag5	0.997 (0.955, 1.041)	1.009 (0.993, 1.026)	1.007 (0.978, 1.036)	1.003 (0.965, 1.043)	1.002 (0.983, 1.020)	0.999 (0.968, 1.030)
lag6	0.996 (0.954, 1.039)	1.003 (0.988, 1.019)	1.000 (0.972, 1.028)	0.999 (0.962, 1.038)	0.984 (0.966, 1.002)	0.995 (0.965,1.026)
lag7	1.008 (0.965, 1.053)	0.992 (0.976, 1.008)	1.007 (0.979, 1.035)	1.013 (0.974, 1.053)	0.975 (0.956, 0.993)	1.010 (0.979, 1.042)
lag01	0.990 (0.929, 1.055)	1.006 (0.980, 1.032)	0.994 (0.956, 1.034)	1.001 (0.950, 1.054)	1.005 (0.979, 1.032)	0.990 (0.948, 1.034)
lag02	0.993 (0.919, 1.073)	1.009 (0.977, 1.042)	1.015 (0.966, 1.067)	1.000 (0.941, 1.063)	0.996 (0.966, 1.027)	1.001 (0.952, 1.053)
lag03	1.036 (0.946, 1.135)	1.017 (0.978, 1.058)	0.999 (0.938, 1.063)	1.017 (0.951, 1.088)	0.990 (0.958, 1.024)	0.984 (0.931, 1.040)
lag04	1.080 (0.976, 1.194)	**1.048 (1.002, 1.095)**	1.002 (0.934, 1.075)	1.025 (0.954, 1.101)	1.002 (0.967, 1.038)	0.976 (0.920, 1.036)
lag05	1.082 (0.972, 1.205)	**1.056 (1.007, 1.107)**	1.002 (0.928, 1.083)	1.023 (0.949, 1.103)	1.007 (0.971, 1.045)	0.973 (0.915, 1.035)
lag06	1.083 (0.966, 1.215)	**1.063 (1.010, 1.119)**	0.987 (0.906, 1.0750	1.017 (0.941, 1.100)	1.002 (0.965, 1.041)	0.966 (0.906, 1.029)
lag07	1.103 (0.976, 1.246)	1.051 (0.995, 1.111)	0.980 (0.895, 1.073)	1.024 (0.944, 1.111)	0.991 (0.952, 1.031)	0.966 (0.904, 1.031)

	CO RR(95%CI)			O_3 RR(95%CI)		
	<18	18-60	>60	<18	18-60	>60
lag0	1.000 (0.998, 1.001)	1.000 (0.999, 1.001)	1.000 (0.999, 1.001)	0.993 (0.967, 1.019)	1.007 (0.996, 1.017)	0.991 (0.971, 1.011)
lag1	0.999 (0.998, 1.001)	1.000 (1.000, 1.001)	1.000 (0.999, 1.001)	1.015 (0.993, 1.038)	1.003 (0.994, 1.013)	0.985 (0.968, 1.004)
lag2	1.000 (0.999, 1.002)	1.000 (1.000, 1.001)	1.001 (1.000, 1.002)	1.018 (0.997, 1.039)	1.007 (0.999, 1.016)	0.988 (0.971, 1.005)
lag3	1.001 (1.000, 1.002)	1.001 (1.000, 1.001)	1.000 (0.999, 1.001)	1.010 (0.990, 1.030)	**1.009 (1.000, 1.017)**	0.995 (0.979, 1.011)
lag4	1.001 (0.999, 1.002)	**1.001 (1.000, 1.001)**	1.000 (0.999, 1.001)	0.997 (0.977, 1.017)	1.005 (0.996, 1.013)	1.000 (0.984, 1.017)
lag5	0.999 (0.998, 1.001)	1.000 (1.000, 1.001)	0.999 (0.999, 1.000)	0.988 (0.968, 1.010)	1.005 (0.996, 1.013)	0.993 (0.976, 1.010)
lag6	1.000 (0.998, 1.001)	1.000 (0.999, 1.000)	0.999 (0.998, 1.001)	0.995 (0.975, 1.016)	1.005 (0.996, 1.014)	0.988 (0.971, 1.005)
lag7	1.000 (0.999, 1.001)	1.000 (0.999, 1.000)	0.999 (0.999, 1.001)	1.001 (0.980, 1.022)	1.007 (0.998, 1.016)	0.993 (0.976, 1.011)
lag01	0.999 (0.997, 1.001)	1.000 (0.999, 1.001)	1.000 (0.998, 1.001)	1.014 (0.982, 1.047)	1.003 (0.988, 1.019)	0.973 (0.946, 1.001)
lag02	1.000 (0.998, 1.002)	1.001 (0.999, 1.002)	1.000 (0.998, 1.001)	1.027 (0.994, 1.061)	1.009 (0.993, 1.025)	0.974 (0.946, 1.003)
lag03	1.001 (0.999, 1.004)	1.001 (1.000, 1.002)	0.999 (0.997, 1.001)	1.028 (0.993, 1.065)	1.013 (0.996, 1.030)	0.978 (0.948, 1.009)
lag04	1.002 (0.999, 1.004)	**1.002 (1.000, 1.003)**	0.999 (0.997, 1.001)	1.019 (0.982, 1.058)	1.012 (0.994, 1.030)	0.984 (0.952, 1.017)
lag05	1.001 (0.998, 1.004)	**1.001 (1.000, 1.003)**	0.999 (0.997, 1.001)	1.003 (0.963, 1.045)	1.011 (0.992, 1.030)	0.981 (0.946, 1.016)
lag06	1.001 (0.998, 1.003)	1.001 (1.000, 1.002)	0.998 (0.996, 1.001)	1.001 (0.957, 1.046)	1.013 (0.992, 1.034)	0.977 (0.940, 1.016)
lag07	1.001 (0.998, 1.004)	1.001 (1.000, 1.002)	0.998 (0.996, 1.000)	1.000 (0.953, 1.050)	1.017 (0.995, 1.041)	0.976 (0.936, 1.017)

Note: statistically significant ($P < 0.05$) are indicated in bold. *P value obtained from the Z-test for the difference of the effect estimates of air pollutants between age groups. No differences between groups were found after Z-test.

Table S10. Relative risk and 95% CI of hospital admissions for mental and behavioral disorders at different lag days for every 10 μg/m^3 increase in pollutants after gender stratification

	$PM_{2.5}$ RR(95%CI)		PM_{10} RR(95%CI)		SO_2 RR(95%CI)	
	female	male	female	male	female	male
lag0	0.998<br>(0.991, 1.006)	0.997<br>(0.989, 1.005)	0.998<br>(0.993, 1.003)	0.996<br>(0.990, 1.001)	1.008<br>(0.994, 1.022)	0.994<br>(0.980, 1.007
lag1	0.999<br>(0.992, 1.006)	0.992<br>(0.985, 1.000)	0.999<br>(0.994, 1.004)	0.992<br>(0.986, 0.998)	0.999<br>(0.985, 1.014)	0.984<br>(0.970, 0.997
lag2	1.002<br>(0.995, 1.008)	1.003<br>(0.996, 1.010)	1.002<br>(0.997, 1.007)	1.000<br>(0.995, 1.006)	1.008<br>(0.993, 1.022)	0.997<br>(0.982, 1.011
lag3	1.001<br>(0.995, 1.008)	1.001<br>(0.994, 1.008)	1.002<br>(0.997, 1.007)	1.002<br>(0.996, 1.007)	1.004<br>(0.990, 1.018)	1.003<br>(0.989, 1.017
lag4	1.003<br>(0.997, 1.010)	1.002<br>(0.995, 1.010)	1.001<br>(0.996, 1.005)	1.001<br>(0.996, 1.006)	1.011<br>(0.997, 1.025)	1.005<br>(0.991, 1.020
lag5	1.005<br>(0.998, 1.011)	1.000<br>(0.993, 1.008)	1.000<br>(0.996, 1.005)	0.999<br>(0.994, 1.004)	1.010<br>(0.996, 1.025)	0.997<br>(0.983, 1.012
lag6	1.001<br>(0.994, 1.008)	1.000<br>(0.993, 1.008)	0.999<br>(0.994, 1.004)	0.999<br>(0.994, 1.004)	1.011<br>(0.997, 1.025)	0.994<br>(0.980, 1.008
lag7	0.994<br>(0.987, 1.001)	1.000<br>(0.992, 1.008)	0.996<br>(0.992,1.002)	0.999<br>(0.994, 1.005)	0.997<br>(0.983, 1.011)	0.994<br>(0.980, 1.008
lag01	1.001<br>(0.991, 1.010)	0.994<br>(0.983, 1.006)	0.999<br>(0.992, 1.006)	0.991<br>(0.983, 0.999)	1.012<br>(0.992, 1.034)	0.983<br>(0.960, 1.008
lag02	1.003<br>(0.992, 1.013)	1.000<br>(0.988, 1.012)	1.001<br>(0.993, 1.008)	0.994<br>(0.985, 1.003)	1.012<br>(0.995, 1.048)	0.984<br>(0.954, 1.014
lag03	1.004<br>(0.993, 1.015)	1.002<br>(0.989, 1.014)	1.003<br>(0.995, 1.011)	0.997<br>(0.988, 1.006)	1.029<br>(0.998, 1.062)	0.988<br>(0.952, 1.025
lag04	1.006<br>(0.994, 1.017)	1.004<br>(0.991, 1.018)	1.003<br>(0.995, 1.011)	0.999<br>(0.989, 1.009)	**1.043**<br>**(1.007, 1.080)**	0.999<br>(0.958, 1.041
lag05	1.008<br>(0.996, 1.020)	1.005<br>(0.991, 1.019)	1.003<br>(0.995, 1.012)	0.999<br>(0.989, 1.009)	**1.056**<br>**(1.017, 1.096)**	1.001<br>(0.957, 1.046
lag06	1.009<br>(0.996, 1.022)	1.006<br>(0.991, 1.021)	1.003<br>(0.994, 1.012)	0.999<br>(0.989, 1.010)	**1.073**<br>**(1.031, 1.117)**	1.005<br>(0.958, 1.054
lag07	1.007<br>(0.993, 1.020)	1.006<br>(0.991, 1.022)	1.002<br>(0.992, 1.012)	0.999<br>(0.987, 1.010)	**1.073**<br>**(1.028, 1.120)**	1.002<br>(0.952, 1.054

	NO_2 RR(95%CI)		CO RR(95%CI)		O_3 RR(95%CI)	
	female	male	female	male	female	male
lag0	1.010 (0.995, 1.025)	0.994 (0.977, 1.011)	1.000 (0.999,1.000)	1.000 (0.999, 1.000)	0.995 (0.986, 1.004)	0.993 (0.984, 1.002)
lag1	1.005 (0.990, 1.020)	0.990 (0.973, 1.007)	1.000 (1.000,1.001)	0.999 (0.999, 1.000)	0.999 (0.991, 1.007)	0.994 (0.986, 1.003)
lag2	1.005 (0.990, 1.020)	1.007 (0.990, 1.024)	1.000 (1.000,1.001)	1.000 (1.000, 1.001)	0.999 (0.991, 1.006)	0.998 (0.990, 1.006)
lag3	0.992 (0.978, 1.006)	1.001 (0.985, 1.018)	1.000 (1.000,1.000)	1.000 (1.000, 1.001)	1.004 (0.997, 1.011)	1.001 (0.994, 1.009)
lag4	1.001 (0.986, 1.016)	1.003 (0.987, 1.020)	1.000 (1.000,1.001)	1.000 (1.000, 1.001)	1.003 (0.995, 1.010)	0.997 (0.989, 1.004)
lag5	1.001 (0.986, 1.015)	0.993 (0.977, 1.010)	1.000 (0.999,1.000)	1.000 (0.999, 1.000)	1.000 (0.993, 1.007)	0.997 (0.989, 1.005)
lag6	0.991 (0.978, 1.005)	0.990 (0.974, 1.006)	1.000 (0.999,1.000)	1.000 (0.999, 1.000)	1.005 (0.997, 1.012)	0.998 (0.990, 1.005)
lag7	0.985 (0.971, 0.999)	0.986 (0.970, 1.002)	1.000 (0.999,1.000)	1.000 (0.999, 1.000)	**1.009 (1.002, 1.017)**	1.006 (0.998, 1.015)
lag01	1.020 (1.000, 1.040)	0.996 (0.974, 1.020)	1.000 (1.000, 1.001)	0.999 (0.998, 1.000)	0.997 (0.985, 1.009)	0.989 (0.975, 1.003)
lag02	1.022 (0.999, 1.046)	1.002 (0.976, 1.030)	1.001 (1.000, 1.001)	1.000 (0.999, 1.001)	0.997 (0.985, 1.010)	0.990 (0.975, 1.004)
lag03	1.017 (0.992, 1.044)	1.004 (0.975, 1.034)	1.001 (1.000, 1.001)	1.000 (0.999, 1.001)	0.998 (0.985, 1.012)	0.993 (0.978, 1.008)
lag04	1.019 (0.991, 1.047)	1.007 (0.975, 1.039)	1.001 (1.000, 1.002)	1.000 (0.999, 1.001)	0.998 (0.984, 1.012)	0.990 (0.974, 1.006)
lag05	1.019 (0.991, 1.048)	1.004 (0.972, 1.038)	1.001 (1.000, 1.002)	1.000 (0.999, 1.001)	0.996 (0.981, 1.011)	0.987 (0.970, 1.005)
lag06	1.016 (0.987, 1.047)	1.002 (0.968, 1.036)	1.001 (0.999, 1.002)	1.000 (0.999, 1.001)	0.997 (0.981,1.014)	0.986 (0.968, 1.005)
lag07	1.011 (0.981, 1.042)	0.995 (0.960, 1.031)	1.000 (0.999, 1.002)	1.000 (0.999, 1.001)	1.003 (0.981,1.021)	0.990 (0.970, 1.011)

Note: statistically significant ($P < 0.05$) are indicated in bold. *P value obtained from the Z-test for the difference of the effect estimates of air pollutants between gender groups. No differences between groups were found after Z-test.

Table S11. Relative risk and 95% CI of depression hospital admissions at different lag days for every 10 μg/m^3 increase in pollutants after gender stratification

	$PM_{2.5}$ RR(95%CI)		PM_{10} RR(95%CI)		SO_2 RR(95%CI)	
	female	male	female	male	female	male
lag0	0.989<br>(0.976, 1.002)	0.994<br>(0.977, 1.012)	0.991<br>(0.982, 0.999)	0.993<br>(0.981, 1.005)	1.000<br>(0.974, 1.027)	0.988<br>(0.953, 1.024)
lag1	0.993<br>(0.981, 1.006)	0.998<br>(0.981, 1.015)	0.995<br>(0.986, 1.004)	0.996<br>(0.984, 1.009)	0.991<br>(0.964, 1.018)	1.006<br>(0.971, 1.043)
lag2	1.003<br>(0.991, 1.014)	1.008<br>(0.992, 1.024)	1.003<br>(0.995, 1.011)	1.002<br>(0.991, 1.014)	1.021<br>(0.994, 1.048)	1.013<br>(0.978, 1.051)
lag3	1.003<br>(0.992, 1.014)	1.002<br>(0.987, 1.018)	1.003<br>(0.995, 1.012)	1.005<br>(0.994, 1.016)	1.014<br>(0.988, 1.041)	0.975<br>(0.941, 1.011)
lag4	1.002<br>(0.991, 1.014)	1.010<br>(0.994, 1.025)	0.997<br>(0.990, 1.005)	1.005<br>(0.995, 1.015)	1.002<br>(0.976, 1.028)	0.986<br>(0.952, 1.021)
lag5	**1.014**<br>**(1.002, 1.025)**	0.999<br>(0.983, 1.015)	1.005<br>(0.998, 1.013)	1.000<br>(0.989, 1.010)	1.019<br>(0.993, 1.046)	0.986<br>(0.952, 1.022)
lag6	**1.012**<br>**(1.000, 1.024)**	1.004<br>(0.988, 1.021)	1.005<br>(0.997, 1.013)	1.001<br>(0.990, 1.012)	**1.041**<br>**(1.015, 1.067)**	0.999<br>(0.965, 1.035)
lag7	0.998<br>(0.986, 1.010)	1.006<br>(0.990, 1.024)	0.998<br>(0.990, 1.006)	1.004<br>(0.992, 1.015)	1.015<br>(0.989, 1.042)	1.004<br>(0.968, 1.040)
lag01	0.991<br>(0.974, 1.007)	0.997<br>(0.973, 1.020)	0.990<br>(0.978, 1.001)	0.993<br>(0.976, 1.009)	0.987<br>(0.950, 1.026)	1.007<br>(0.954, 1.062)
lag02	0.996<br>(0.979, 1.014)	1.005<br>(0.980, 1.031)	0.994<br>(0.982, 1.007)	0.996<br>(0.978, 1.014)	1.006<br>(0.959, 1.055)	1.027<br>(0.960, 1.097)
lag03	1.000<br>(0.981, 1.019)	1.007<br>(0.980, 1.034)	0.998<br>(0.984, 1.011)	1.000<br>(0.981, 1.020)	1.025<br>(0.968, 1.085)	1.008<br>(0.931, 1.092)
lag04	1.002<br>(0.982, 1.022)	1.014<br>(0.986, 1.042)	0.997<br>(0.982, 1.011)	1.004<br>(0.984, 1.025)	1.026<br>(0.962, 1.094)	0.998<br>(0.912, 1.091)
lag05	1.011<br>(0.990, 1.032)	1.013<br>(0.984, 1.042)	1.001<br>(0.986, 1.016)	1.005<br>(0.984, 1.026)	1.049<br>(0.980, 1.123)	1.009<br>(0.9181,1101)
lag06	1.017<br>(0.996, 1.039)	1.016<br>(0.986, 1.047)	1.005<br>(0.989, 1.021)	1.007<br>(0.985, 1.029)	**1.100**<br>**(1.023, 1.182)**	1.024<br>(0.926, 1.133)
lag07	1.018<br>(0.995, 1.041)	1.019<br>(0.987, 1.052)	1.005<br>(0.989, 1.021)	1.008<br>(0.984, 1.032)	**1.121**<br>**(1.038, 1.211)**	1.035<br>(0.929, 1.152)

	NO_2 RR(95%CI)		CO RR(95%CI)		O_3 RR(95%CI)	
	female	male	female	male	female	male
lag0	1.009 (0.984, 1.035)	0.987 (0.952, 1.022)	1.000 (0.999, 1.000)	1.000 (0.998, 1.001)	0.988 (0.971, 1.004)	1.007 (0.986, 1.029)
lag1	0.999 (0.973, 1.026)	1.002 (0.966, 1.039)	1.000 (0.999, 1.000)	1.000 (0.998, 1.001)	0.996 (0.982, 1.010)	0.996 (0.977, 1.016)
lag2	1.016 (0.990, 1.042)	1.028 (0.992, 1.066)	1.000 (0.999, 1.001)	1.000 (0.999, 1.002)	0.989 (0.976, 1.002)	0.986 (0.968, 1.004)
lag3	0.998 (0.974, 1.023)	0.992 (0.959, 1.027)	1.000 (0.999, 1.001)	0.999 (0.998, 1.001)	1.007 (0.994, 1.019)	0.988 (0.970, 1.006)
lag4	0.990 (0.965, 1.015)	0.995 (0.961, 1.030)	1.000 (0.999, 1.000)	1.000 (0.999, 1.001)	1.006 (0.994, 1.019)	0.983 (0.965, 1.000)
lag5	0.999 (0.974, 1.024)	0.980 (0.947, 1.014)	1.000 (0.999, 1.001)	0.999 (0.998, 1.001)	1.001 (0.988, 1.015)	0.987 (0.969, 1.005)
lag6	1.006 (0.982, 1.030)	0.992 (0.960, 1.026)	1.000 (1.000, 1.001)	1.000 (0.999, 1.001)	1.004 (0.991, 1.017)	0.992 (0.974, 1.010)
lag7	0.988 (0.964, 1.013)	0.981 (0.948, 1.016)	1.001 (0.999, 1.001)	1.000 (0.999, 1.001)	**1.018 (1.005, 1.031)**	1.018 (1.000, 1.037)
lag01	1.017 (0.984, 1.052)	1.001 (0.954, 1.051)	1.000 (0.998, 1.001)	1.000 (0.998, 1.001)	0.988 (0.968, 1.008)	0.998 (0.970, 1.027)
lag02	1.033 (0.993, 1.073)	1.028 (0.971, 1.087)	1.000 (0.998, 1.001)	1.000 (0.998, 1.002)	0.983 (0.963, 1.004)	0.987 (0.958, 1.017)
lag03	1.033 (0.989, 1.078)	1.024 (0.962, 1.089)	1.000 (0.998, 1.001)	1.000 (0.997, 1.002)	0.987 (0.966, 1.009)	0.978 (0.948, 1.010)
lag04	1.027 (0.980, 1.075)	1.021 (0.956, 1.091)	1.000 (0.998, 1.001)	1.000 (0.997, 1.002)	0.990 (0.967, 1.014)	0.967 (0.934, 1.000)
lag05	1.029 (0.980, 1.080)	1.012 (0.945, 1.085)	1.000 (0.998, 1.002)	0.999 (0.997, 1.002)	0.990 (0.965, 1.016)	0.958 (0.923, 0.994)
lag06	1.035 (0.985, 1.088)	1.013 (0.943, 1.087)	1.000 (0.999, 1.002)	1.000 (0.997, 1.002)	0.992 (0.965, 1.020)	0.952 (0.914, 0.991)
lag07	1.033 (0.980, 1.088)	1.007 (0.935, 1.085)	1.001 (0.999, 1.002)	1.000 (0.997, 1.002)	1.002 (0.972, 1.032)	0.957 (0.917, 0.999)

Note: statistically significant ($P < 0.05$) are indicated in bold. *P value obtained from the Z-test for the difference of the effect estimates of air pollutants between gender groups. No differences between groups were found after Z-test.

Table S12. Relative risk and 95% CI of hospital admissions for anxiety disorders at different lag days for every 10 μg/m^3 increase in pollutants after gender stratification

	$PM_{2.5}$ RR(95%CI)		PM_{10} RR(95%CI)		SO_2 RR(95%CI)	
	female	male	female	male	female	male
lag0	1.001 (0.992, 1.011)	1.000 (0.990, 1.011)	1.000 (0.994, 1.006)	0.999 (0.992, 1.007)	1.011 (0.993, 1.029)	0.982 (0.964, 1.001)
lag1	1.004 (0.995, 1.013)	0.998 (0.987, 1.008)	1.001 (0.995, 1.008)	0.995 (0.988, 1.003)	1.007 (0.988, 1.025)	0.976 (0.958, 0.995)
lag2	1.004 (0.995, 1.012)	1.007 (0.997, 1.017)	1.002 (0.996, 1.008)	1.002 (0.995, 1.009)	1.006 (0.987, 1.025)	0.993 (0.974, 1.013)
lag3	1.003 (0.995, 1.012)	1.002 (0.993, 1.012)	1.003 (0.997, 1.009)	1.001 (0.994, 1.008)	1.006 (0.988, 1.024)	1.006 (0.987, 1.025)
lag4	1.007 (0.998, 1.015)	1.005 (0.994, 1.015)	1.003 (0.997, 1.009)	1.003 (0.996, 1.010)	**1.028 (1.009, 1.047)**	1.005 (0.986, 1.025)
lag5	1.002 (0.993, 1.011)	1.003 (0.993, 1.013)	0.998 (0.992, 1.004)	1.002 (0.995, 1.009)	1.008 (0.989, 1.027)	0.997 (0.977, 1.018)
lag6	0.996 (0.987, 1.006)	1.000 (0.990, 1.011)	0.996 (0.989, 1.002)	0.999 (0.992, 1.006)	1.004 (0.986, 1.023)	0.990 (0.971, 1.009)
lag7	0.991 (0.982, 1.001)	0.996 (0.985, 1.007)	0.995 (0.988, 1.002)	0.996 (0.988, 1.003)	0.989 (0.973, 1.006)	0.981 (0.961, 1.000)
lag01	1.005 (0.993, 1.018)	0.995 (0.980, 1.010)	1.002 (0.993, 1.011)	0.992 (0.981, 1.003)	1.020 (0.993, 1.047)	0.969 (0.937, 1.001)
lag02	1.006 (0.993, 1.020)	1.000 (0.984, 1.016)	1.004 (0.994, 1.014)	0.994 (0.982, 1.006)	1.025 (0.991, 1.059)	0.969 (0.930, 1.010)
lag03	1.008 (0.994, 1.022)	1.001 (0.984, 1.018)	1.006 (0.995, 1.017)	0.995 (0.982, 1.008)	1.030 (0.989, 1.072)	0.983 (0.935, 1.033)
lag04	1.012 (0.996, 1.027)	1.003 (0.984, 1.021)	1.008 (0.997, 1.019)	0.997 (0.984, 1.011)	**1.063 (1.016, 1.112)**	1.003 (0.948, 1.060)
lag05	1.012 (0.996, 1.028)	1.004 (0.985, 1.023)	1.007 (0.996, 1.019)	0.999 (0.985, 1.013)	**1.072 (1.021, 1.124)**	1.006 (0.948, 1.069)
lag06	1.010 (0.994, 1.027)	1.004 (0.984, 1.024)	1.005 (0.993, 1.018)	0.998 (0.984, 1.013)	**1.078 (1.023, 1.136)**	1.013 (0.950, 1.080)
lag07	1.006 (0.989, 1.024)	1.001 (0.980, 1.022)	1.003 (0.990, 1.017)	0.995 (0.979, 1.011)	**1.069 (1.011, 1.131)**	0.995 (0.928, 1.066)

	NO_2 RR(95%CI)		CO RR(95%CI)		O_3 RR(95%CI)	
	female	male	female	male	female	male
lag0	1.010 (0.990, 1.029)	1.000 (0.978, 1.023)	1.000 (0.999, 1.001)	1.000 (0.999, 1.001)	0.996 (0.984, 1.008)	0.997 (0.984, 1.010)
lag1	1.010 (0.990, 1.031)	0.995 (0.972, 1.018)	1.001 (1.000, 1.001)	0.999 (0.999, 1.000)	1.002 (0.992, 1.012)	0.999 (0.987, 1.010)
lag2	0.998 (0.978, 1.017)	1.007 (0.984, 1.030)	1.000 (1.000, 1.001)	1.000 (1.000, 1.001)	1.007 (0.997, 1.016)	1.004 (0.994, 1.015)
lag3	0.992 (0.973, 1.011)	1.001 (0.979, 1.023)	1.000 (1.000, 1.001)	1.000 (1.000, 1.001)	1.005 (0.996, 1.014)	1.007 (0.997, 1.018)
lag4	1.015 (0.995, 1.036)	1.005 (0.982, 1.028)	**1.001 (1.000, 1.001)**	1.001 (1.000, 1.001)	1.003 (0.994, 1.012)	1.003 (0.992, 1.013)
lag5	1.009 (0.990, 1.029)	0.999 (0.977, 1.022)	1.000 (0.999, 1.001)	1.000 (0.999, 1.001)	0.997 (0.987, 1.007)	1.003 (0.992, 1.014)
lag6	0.988 (0.969, 1.007)	0.994 (0.973, 1.016)	1.000 (0.999, 1.001)	1.000 (0.999, 1.000)	0.999 (0.989, 1.010)	1.000 (0.990, 1.011)
lag7	0.987 (0.968, 1.007)	0.983 (0.961, 1.006)	1.000 (0.999, 1.001)	1.000 (0.999, 1.000)	1.000 (0.989, 1.011)	1.008 (0.997, 1.020)
lag01	1.015 (0.988, 1.042)	0.996 (0.965, 1.028)	1.001 (1.000, 1.002)	0.999 (0.998, 1.000)	1.003 (0.987, 1.020)	0.991 (0.972, 1.011)
lag02	1.006 (0.975, 1.038)	0.996 (0.960, 1.033)	1.001 (1.000, 1.002)	1.000 (0.998, 1.001)	1.011 (0.994, 1.028)	0.997 (0.977, 1.018)
lag03	0.999 (0.966, 1.034)	0.994 (0.954, 1.035)	1.001 (1.000, 1.002)	1.000 (0.999, 1.002)	1.013 (0.995, 1.031)	1.004 (0.983, 1.026)
lag04	1.009 (0.973, 1.046)	0.997 (0.954, 1.041)	**1.002 (1.000, 1.003)**	1.001 (0.999, 1.002)	1.012 (0.994, 1.031)	1.004 (0.981, 1.027)
lag05	1.015 (0.978, 1.054)	0.998 (0.954, 1.045)	1.001 (1.000, 1.003)	1.000 (0.999, 1.002)	1.007 (0.987, 1.027)	1.004 (0.980, 1.028)
lag06	1.010 (0.971, 1.050)	0.997 (0.951, 1.044)	1.001 (1.000, 1.002)	1.000 (0.999, 1.002)	1.006 (0.984, 1.028)	1.006 (0.980, 1.033)
lag07	1.003 (0.964, 1.045)	0.986 (0.939, 1.035)	1.001 (0.999, 1.002)	1.000 (0.998, 1.002)	1.007 (0.984, 1.031)	1.011 (0.983, 1.040)

Note: statistically significant ($P < 0.05$) are indicated in bold. *P value obtained from the Z-test for the difference of the effect estimates of air pollutants between gender groups. No differences between groups were found after Z-test.

Table S13. Relative risk and 95% CI of hospital admissions for mental and behavioral disorders per 10 μg/m^3 increase in pollutants using different of freedom per year

df for time (per year)	$PM_{2.5}$	PM_{10}	SO_2	NO_2	CO	O_3
Total						
5	1.003 (0.997,1.009)	1.003 (0.998,1.007)	1.011 (0.998,1.024)	1.006 (0.992,1.020)	1.000 (1.000.1.001)	1.010 (1.003,1.016)
6	1.003 (0.997,1.009)	1.003 (0.998,1.007)	1.010 (0.998,1.024)	1.006 (0.992,1.020)	1.000 (1.000.1.001)	1.010 (1.003,1.016)
7	1.003 (0.997,1.009)	1.003 (0.998,1.007)	1.010 (0.998,1.024)	1.006 (0.992,1.020)	1.000 (1.000.1.001)	1.010 (1.003,1.016)
8	1.003 (0.997,1.009)	1.003 (0.998,1.007)	1.010 (0.998,1.024)	1.006 (0.992,1.020)	1.000 (1.000.1.001)	1.010 (1.003,1.016)
9	1.003 (0.997,1.009)	1.003 (0.998,1.007)	1.010 (0.998,1.024)	1.006 (0.992,1.020)	1.000 (1.000.1.001)	1.010 (1.003,1.016)
10	1.003 (0.997,1.009)	1.003 (0.998,1.007)	1.010 (0.998,1.024)	1.006 (0.992,1.020)	1.000 (1.000.1.001)	1.010 (1.003,1.016)
Depression						
5	1.010 (1.000,1.020)	1.006 (0.999, 1.013)	1.030 (1.008, 1.053)	1.019 (0.996, 1.042)	1.001 (1.000.1.001)	1.020 (1.008, 1.032)
6	1.011 (1.001,1.021)	1.006 (0.999, 1.013)	1.032 (1.010, 1.055)	1.022 (0.999, 1.046)	1.001 (1.000.1.001)	1.019 (1.008, 1.031)
7	1.011 (1.001,1.021)	1.006 (0.999, 1.013)	1.032 (1.010, 1.055)	1.022 (1.000, 1.046)	1.001 (1.000.1.001)	1.020 (1.008, 1.031)
8	1.011 (1.001,1.021)	1.006 (0.999, 1.013)	1.032 (1.010, 1.055)	1.023 (1.000, 1.046)	1.001 (1.000.1.001)	1.020 (1.008, 1.031)
9	1.012 (1.002,1.022)	1.007 (1.000, 1.014)	1.032 (1.010, 1.055)	1.026 (1.003, 1.049)	1.001 (1.000.1.001)	1.019 (1.008, 1.031)
10	1.012 (1.002,1.022)	1.007 (1.000, 1.014)	1.032 (1.010, 1.055)	1.026 (1.003, 1.049)	1.001 (1.000.1.001)	1.020 (1.008, 1.031)
Anxiety						
5	1.007 (1.000, 1.015)	1.003 (0.998,1.008)	1.022 (1.007,1.037)	1.013 (0.996,1.031)	1.001 (1.000.1.001)	1.006 (0.998,1.031)
6	1.007 (1.000, 1.015)	1.003 (0.998,1.008)	1.022 (1.007,1.037)	1.013 (0.996,1.031)	1.001 (1.000.1.001)	1.006 (0.998,1.031)
7	1.007 (1.000, 1.015)	1.003 (0.998,1.008)	1.022 (1.007,1.037)	1.013 (0.996,1.031)	1.001 (1.000.1.001)	1.006 (0.998,1.031)
8	1.007 (1.000, 1.015)	1.003 (0.998,1.008)	1.022 (1.007,1.037)	1.013 (0.996,1.031)	1.001 (1.000.1.001)	1.006 (0.998,1.031)
9	1.007 (1.000, 1.015)	1.003 (0.998,1.008)	1.022 (1.007,1.037)	1.013 (0.996,1.031)	1.001 (1.000.1.001)	1.006 (0.998,1.031)
10	1.007 (1.000, 1.015)	1.003 (0.998,1.008)	1.022 (1.007,1.037)	1.013 (0.996,1.031)	1.001 (1.000.1.001)	1.006 (0.998,1.031)

Table S14. Relative risk and 95%CI of mental and behavioral disorders from exposure to pollutants in two-pollutant models

adjust variables	Total	Depression	Anxiety
$PM_{2.5}$	1.006(0.999,1.012)	**1.011(1.001,1.021)**	1.007(1.000,1.015)
$+PM_{10}$	1.004(0.998, 1.010)	**1.012(1.002, 1.022)**	1.008(1.000, 1.015)
$+SO_2$	1.004(0.997, 1.009)	**1.012(1.002, 1.022)**	1.007(1.000, 1.015)
$+NO_2$	1.003(0.997, 1.009)	**1.012(1.002, 1.022)**	1.007(1.000, 1.015)
+CO	1.004(0.997, 1.010)	**1.012(1.002, 1.022)**	1.007(1.000, 1.015)
$+O_3$	1.004(0.998, 1.010)	**1.012(1.002, 1.022)**	1.005(0.997, 1.015)
PM_{10}	1.003(0.999,1.008)	1.006(0.999,1.013)	1.003(0.998,1.008)
$+PM_{2.5}$	1.003(0.999, 1.007)	1.006(0.999, 1.012)	1.003(0.997, 1.008)
$+SO_2$	1.003(0.997, 1.007)	1.006(0.999, 1.013)	1.003(0.998, 1.008)
$+NO_2$	1.003(0.999, 1.007)	1.006(0.999, 1.013)	1.003(0.998, 1.008)
+CO	1.003(0.999, 1.007)	1.006(0.999, 1.013)	1.003(0.998, 1.008)
$+O_3$	1.003(0.999, 1.007)	1.006(1.000, 1.013)	1.003(0.998, 1.008)
SO_2	**1.016(1.002,1.031)**	**1.032(1.010,1.055)**	**1.022(1.007,1.037)**
$+PM_{2.5}$	1.011(0.998, 1.024)	**1.035(1.012, 1.058)**	**1.022(1.007, 1.037)**
+PM10	1.011(0.998, 1.024)	**1.034(1.012, 1.057)**	**1.022(1.007,1.037)**
$+NO_2$	1.010(0.997, 1.024)	**1.034(1.011, 1.057)**	**1.022(1.007, 1.037)**
+CO	1.011(0.998, 1.024)	**1.034(1.012, 1.057)**	**1.022(1.007, 1.037)**
$+O_3$	1.010(0.998, 1.024)	**1.033(1.012, 1.056)**	**1.022(1.007,1.037)**
NO_2	1.007(0992, 1.023)	1.022(0.999,1.046)	1.013(0.996,1.031)
$+PM_{2.5}$	1.008(0.994, 1.022)	**1.025(1.002, 1.049)**	1.003(0.985, 1.021)
+PM10	1.009(0.995, 1.023)	**1.026(1.003, 1.049)**	1.004(0.986, 1.022)
$+SO_2$	1.006(0.993, 1.021)	**1.023(1.000, 1.046)**	1.003(0.985, 1.021)
+CO	1.004(0.990, 1.018)	**1.023(1.000, 1.046)**	1.003(0.985, 1.021)
$+O_3$	1.004(0.991, 1.018)	**1.025(1.002, 1.048)**	1.003(0.985, 1.021)
CO	1.000(1.000, 1.001)	1.001(1.000,1.001)	**1.001(1.000,1.001)**
$+PM_{2.5}$	1.000(1.000, 1.001)	1.001(0.999, 1.001)	**1.001(1.000, 1.001)**
+PM10	1.000(1.000, 1.001)	1.000(0.999, 1.001)	**1.001(1.000, 1.001)**
$+SO_2$	1.000(1.000, 1.001)	1.000(0.999, 1.001)	**1.001(1.000, 1.001)**
$+NO_2$	1.000(1.000, 1.001)	1.000(0.999, 1.001)	**1.001(1.000, 1.001)**
$+O_3$	1.000(0.999, 1.001)	1.000(0.999, 1.001)	**1.000(1.000, 1.001)**
O_3	**1.010(1.002,1.018)**	**1.019(1.008,1.031)**	1.004(0.995,1.013)
$+PM_{2.5}$	1,011(1.004, 1.017)	**1.020(1.008, 1.032)**	1.006(0.997, 1.014)
+PM10	1,011(1.004, 1.017)	**1.020(1.008, 1.032)**	1.005(0.997, 1.014)
$+SO_2$	1,011(1.004, 1.017)	**1.021(1.009, 1.033)**	1.006(0.997, 1.014)
$+NO_2$	0.999(1.004, 1.018)	**1.021(1.009, 1.033)**	1.006(0.997, 1.014)
+CO	1.004(0.998, 1.011)	**1.021(1.009,1.033)**	1.006(0.997, 1.015)

Note: statistically significant ($P < 0.05$) are indicated in bold.
